# The Role of T1-Weighted Derived Measures of Neurodegeneration for Assessing Disability Progression in Multiple Sclerosis

**DOI:** 10.3389/fneur.2017.00433

**Published:** 2017-09-04

**Authors:** Maria A. Rocca, Giancarlo Comi, Massimo Filippi

**Affiliations:** ^1^Neuroimaging Research Unit, Institute of Experimental Neurology, San Raffaele Scientific Institute, Vita-Salute San Raffaele University, Milan, Italy; ^2^Department of Neurology, Institute of Experimental Neurology, San Raffaele Scientific Institute, Vita-Salute San Raffaele University, Milan, Italy

**Keywords:** multiple sclerosis, disability, neurodegeneration, magnetic resonance imaging, atrophy, black holes

## Abstract

**Introduction:**

Multiple sclerosis (MS) is characterised by the accumulation of permanent neurological disability secondary to irreversible tissue loss (neurodegeneration) in the brain and spinal cord. MRI measures derived from T1-weighted image analysis (i.e., black holes and atrophy) are correlated with pathological measures of irreversible tissue loss. Quantifying the degree of neurodegeneration *in vivo* using MRI may offer a surrogate marker with which to predict disability progression and the effect of treatment. This review evaluates the literature examining the association between MRI measures of neurodegeneration derived from T1-weighted images and disability in MS patients.

**Methods:**

A systematic PubMed search was conducted in January 2017 to identify MRI studies in MS patients investigating the relationship between “black holes” and/or atrophy in the brain and spinal cord, and disability. Results were limited to human studies published in English in the previous 10 years.

**Results:**

A large number of studies have evaluated the association between the previous MRI measures and disability. These vary considerably in terms of study design, duration of follow-up, size, and phenotype of the patient population. Most, although not all, have shown that there is a significant correlation between disability and black holes in the brain, as well as atrophy of the whole brain and grey matter. The results for brain white matter atrophy are less consistently positive, whereas studies evaluating spinal cord atrophy consistently showed a significant correlation with disability. Newer ways of measuring atrophy, thanks to the development of segmentation and voxel-wise methods, have allowed us to assess the involvement of strategic regions of the CNS (e.g., thalamus) and to map the regional distribution of damage. This has resulted in better correlations between MRI measures and disability and in the identification of the critical role played by some CNS structures for MS clinical manifestations.

**Conclusion:**

The evaluation of MRI measures of atrophy as predictive markers of disability in MS is a highly active area of research. At present, measurement of atrophy remains within the realm of clinical studies, but its utility in clinical practice has been recognized and barriers to its implementation are starting to be addressed.

## Introduction

The application of magnetic resonance imaging (MRI) techniques is integral to our understanding of the pathogenesis of multiple sclerosis (MS). Historically, MS was considered to be an autoimmune-driven inflammatory disease characterised by focal white matter (WM) demyelination (1), visualized as gadolinium-enhancing T1 and hyperintense T2 lesions on conventional MRI scans (2). However, the correlation between conventional MRI measures and the extent of clinical disability is limited, particularly when applied to individual patients—a phenomenon known as the “clinical–MRI paradox” (3).

Two major advances in our understanding of MS are helping to resolve this paradox. First, the pathological substrates of MS extend well beyond focal WM lesions. Advanced MRI techniques, as well as postmortem histopathological findings, have shown that MS is also characterised by more widespread damage to the so-called “normal-appearing” WM (NAWM), as well as focal and diffuse damage to the grey matter (GM) of the brain and spinal cord (3, 4). Second, it is also now apparent that at least some of the neurodegenerative changes in MS are independent of inflammatory demyelination (5, 6). Indeed, a number of pathogenic changes have been suggested to drive neurodegeneration, including mitochondrial damage, iron deposition, microglial activation, and altered ion channel activity (7–9).

It is well accepted that neurodegenerative changes, irrespective of their aetiology, underlie the accumulation of permanent neurological disability that characterises MS (10–13). As a consequence, a key area of research in the field of MS is the evaluation of neurodegenerative changes using MRI techniques and their association with clinical disability and cognitive dysfunction. The ultimate aim is to find predictive biomarkers for neurodegeneration and disability and to develop sensitive and specific imaging markers that can be used to monitor disease progression and evaluate the response to treatment.

The aim of this review was to discuss the literature examining the association between MRI measures of neurodegeneration derived from the analysis of T1-weighted images (black holes and atrophy) and disability in patients with MS. Black holes represent areas of focal axonal damage and irreversible tissue destruction (14), while atrophy is a more widespread axonal loss that is thought to be caused by tissue damage within lesions and Wallerian degeneration in related fibre pathways (15). At the level of the GM, neuronal loss and shrinkage also contribute to atrophy (16).

Atrophy is most commonly quantified as the loss of overall brain tissue from T1-weighted images. Some methods for atrophy quantification also work on T2 and FLAIR images. Advances in the methods of analysis have made it possible to measure atrophy of spinal cord, GM, WM, and specific regions and structures of the brain (e.g., thalamus, hippocampus, etc.). A number of techniques are used to quantify atrophy, ranging from manual bi-dimensional assessment to automated or semi-automated volumetric measurement. Methods for the quantification of atrophy are continuously evolving, improving not only image acquisition and analysis strategies but also in terms of increased understanding of the technical (e.g., sequence geometry, WM lesion influence, etc.), physiological (e.g., age, sex, hydration, etc.), lifestyle (e.g., alcohol consumption, smoking, diet), genetics (e.g., apolipoprotein E expression), and other factors (e.g., diabetes, cardiovascular risks) that may affect brain volume results. For instance, it is now established that WM lesions affect atrophy calculations, since they influence the detection of GM/WM/cerebrospinal fluid intensity differences. Different techniques, that can be applied in a wide range of atrophy tools, have been proposed to fill in the signal from these lesions and alleviate this problem. For a review of these techniques and factors, the reader is referred to review articles on this topic (17–20). With regards to the measurement of disability in patients with MS, a number of scales and tests are available. The most commonly used (at least in clinical trials) is the Expanded Disability Status Scale (EDSS), which is measured on a scale of 0 (no disability) to 10 (death due to MS) (21). Others include the timed 25-foot walk (T25FW) (22), the 9-hole peg test (9HPT) (23), and the Multiple Sclerosis Functional Composite (MSFC) (24), which is a composite of the T25FW, 9HPT, and a test of cognitive function. Although of significant interest, studies focusing on the association between atrophy and cognitive dysfunction were beyond the scope of this review and the reader is referred to a recent comprehensive review of the topic (25).

## Methods

A systematic PubMed search was conducted in January 2017 to identify studies investigating the relationship between disability and black holes and/or atrophy in the brain and spinal cord. Results were limited to human studies published in English in the previous 10 years. Studies that examined the effect of disease-modifying treatments on measures of neurodegeneration were excluded, as this was outside the scope of this review. The search terminology is summarized in the Table S1 in Supplementary Material.

Results are presented first for studies that assessed black holes/atrophy in the brain, followed by studies that assessed these measures in the spinal cord. Studies that assessed both brain and spinal cord atrophy in relation to disability are presented in a separate section.

## Results

### Brain Black Holes and Atrophy

Overall, 59 key studies evaluating the association between MRI measures of brain neurodegeneration and disability were identified (Table 1). Of these, 38 were cross-sectional and 21 were longitudinal in design (either for clinical or MRI variables). Most were non-phenotype specific (*n* = 35), while 10 focused on relapsing-remitting multiple sclerosis (RRMS), four on primary progressive multiple sclerosis (PPMS), three on relapsing MS, two on clinically isolated syndrome (CIS), and one on relapsing onset (i.e., CIS, RRMS, and secondary progressive) MS. The remaining four studies compared MS subtypes. In the majority of studies, the EDSS score was used to assess disability; other disability measures included the MSFC, the Multiple Sclerosis Severity Score (MSSS), the 9HPT, and the T25FW. Most of the studies evaluated whole brain atrophy as a measure of neurodegeneration; other measures included black holes and atrophy of the global GM, global WM, regional GM, and/or GM of specific structures.

**Table 1:** MRI studies evaluating the relationship between brain black holes/atrophy and disability.

(a) Cross-sectional studies					
Reference	Patients (*n*)		MRI measure of neurodegeneration [acquisition/quantification methods]	Measure of disability	Results
**CIS**					
Fisniku et al. (26)^a^	73		GM fraction WM fraction [3D T1w/SPM]	EDSS MSFC	GM fraction, but not WM fraction, correlated with EDSS (*r* = −0.48; *p* < 0.001) and MSFC (*r* = 0.59; *p* < 0.001) GM fraction explained more of the variability in clinical measures than did WM lesion load
Audoin et al. (27)	62		Regional GM atrophy GM atrophy of specific structures [3D T1w/VBM]	EDSS	Significant correlation between EDSS and atrophy of the right cerebellum (*r* = −0.37; *p* = 0.0027)
**RRMS**					
Prinster et al. (28)	128		Global GM volume Global WM volume Regional GM volume [T1w and PD-T2w/VBM]	EDSS	No significant correlation between global GM loss and EDSS Significant correlation between global WM loss and EDSS (*p* < 0.0001) Significant linear correlation between regional bilateral GM loss and EDSS in the primary motor and somatosensory areas and the middle frontal gyri, with extension to the right middle temporal gyrus
Riccitelli et al. (29)	78		Regional WM atrophy Regional GM atrophy [3D T1w/VBM]	EDSS	In patients with EDSS scores ≤3.0, WM atrophy was restricted to a few WM tracts; in those with EDSS scores >3.0, several tracts of the cerebral and cerebellar hemispheres were involved. In patients with EDSS scores >3.0, regions with more severe GM atrophy were the left basal ganglia and thalamus and the right precentral gyrus
Nygaard et al. (30)	61		Cortical surface area, thickness and volume [3D T1w/FreeSurfer]	EDSS	No significant correlation between EDSS and cortical surface area, thickness, or volume
Hasan et al. (31)	54		Regional volume-to-intracranial volume % of a wide range of GM and WM structures [3D T1w/FreeSurfer]	EDSS	Significant correlations between EDSS and % volume of frontal lobe WM (*r* = 0.286; *p* = 0.04), CLWM (*r* = 0.28; *p* = 0.045), insular WM (*r* = 0.301; *p* = 0.03), entire corpus callosum (*r* = 0.411; *p* = 0.002), periventricular WM (*r* = 0.279; *p* = 0.045), anterior corpus callosum (*r* = 0.37; *p* = 0.01), middle anterior corpus callosum (*r* = 0.35; *p* = 0.01), truncus corpus callosum (*r* = 0.32; *p* = 0.02), corpus callosum isthmus (*r* = 0.30; *p* = 0.03), and corpus callosum splenium (*r* = 0.31; *p* = 0.03)
Duan et al. (32)	26		Global GM volume [3D T1w/VBM and SPM]	EDSS	No correlation between GM loss and EDSS
Mesaros et al. (33)	28 (pediatric)		Regional GM loss [T1w conventional spin-echo/SIENAX]	EDSS	No correlation between thalamic GM loss and disability
Llufriu et al. (34)	21		Corpus callosum area (total) Corpus callosum area (segments 1–7) Corpus callosum volume [3D T1w/SIENAX]	EDSS MSFC	Area of segment 1 of corpus callosum correlated with EDSS (*r* = −0.442; *p* = 0.045) No significant correlation between other corpus callosum measures and disability
**RELAPSING**					
Tao et al. (35)	88		Deep GM atrophy [3D T1w/TBM]	EDSS	Significant correlation between EDSS and atrophy of the thalamus (*r* = −0.51), caudate nucleus (*r* = −0.43), and putamen (*r* = −0.36) (*p* < 0.0001 for all)
**RELAPSING ONSET**					
D’Ambrosio et al. (36)	95		Whole brain volume GM volume WM volume Cerebellar volume (total, anterior, posterior) [3D T1w/SIENAX and SPM]	EDSS 9HPT	Significant correlation between the EDSS and all cerebellar volumes; only anterior cerebellar volume remained significant in multivariate analysis (beta coefficient, −0.320; *p* = 0.003) Significant correlation between the 9HPT and whole brain volume and all cerebellar volumes; only anterior cerebellar volume remained significant in multivariate analysis (beta coefficient, 0.264; *p* = 0.02)
**REMITTING**					
Mineev et al. (37)	65		Brain atrophy (cerebral parenchymal volume) [Semiautomatic computer program]	FSS EDSS	Significant correlations between brain atrophy and EDSS and FSS for pelvic dysfunction (*r* = −0.36; *p* < 0.05)
**PPMS**					
Bodini et al. (38)	35		Regional GM volume [3D T1w/VBM]	EDSS MSFC subtests	Patients with greater GM atrophy in the right sensory-motor cortex had greater upper limb disability measured using 9HPT (coefficient = 1.27; *p* = 0.01) No correlation between GM atrophy and predefined EDSS groups (EDSS score ≤3.5; 4–5.5; ≥6)
Galego et al. (39)	19		Volumes of: Neocortex Total WM Total subcortical GM Putamen, caudate, globus pallidus, thalamus, hippocampus, brainstem, corpus callosum, and precentral gyrus [3D T1w/FreeSurfer]	EDSS	No correlation between EDSS and any of the GM or WM structures analyzed
**MS**					
Roosendaal et al. (40)	927		GM volume WM volume [3D T1w/SIENAX]	EDSS	Significant correlation between EDSS and GM volume (OR = 0.67; *p* < 0.001), but not WM volume
Steenwijk et al. (41)	208		Global cortical thickness [3D T1w/SIENAX]	EDSS	Reduced cortical thickness was one of the significant predictors of EDSS in a multivariate model (beta = −0.227; *p* < 0.001)
Howard et al. (42)	194		Brain volume Global WM volume Global GM volume [3D T1w/SIENAX]	Need for ambulatory assistance	Significant difference in brain volume (*p* = 0.001), GM volume (*p* = 0.0008), and WM volume (*p* = 0.02) in those requiring ambulatory assistance vs those who did not
Tauhid et al. (43)	175		Brain atrophy [T2w dual echo/BPF]	EDSS	Data were analyzed according to four phenotypes: Type 1, low T2LV/mild atrophy; Type 2, high T2LV/mild atrophy; Type 3, low T2LV/high atrophy; Type 4, high T2LV/high atrophy Significant correlation between BPF and EDSS for overall population (*r* = −0.57; *p* < 0.0001) and Type 4 patients (*r* = −0.46; *p* < 0.0001)
Preziosa et al. (44)	172		Cerebellar WM and GM volumes [3D T1w/SPM and SIENAX]	Patients categorized according to degree of disability: EDSS scores <4.0 or ≥4.0 Cerebellar FSS = 0 or ≥1 Brainstem FSS = 0 or ≥1	Significantly lower cerebellar GM volume in patients with disability according to EDSS (*p* = 0.01) and cerebellar FSS (*p* = 0.006) Significantly lower cerebellar WM volume in patients with disability according to EDSS (*p* = 0.03) and brainstem FSS (*p* = 0.004)
Yaldizli et al. (45)	146		Olfactory bulb volume [3D T1w/AMIRA]	EDSS	No correlation between olfactory bulb volume and EDSS
Calabrese et al. (46)	115		Global and regional cortical thickness [3D T1w/BPF and Freesurfer]	EDSS FSS	No correlation between mean cortical thinning and EDSS in patients with possible or definite MS Significant correlation between motor FSS and precentral gyrus thinning in both groups (*r* = −0.487, *p* = 0.006 for possible MS; *r* = −0.626, *p* < 0.001 for definite MS) Significant correlation between visual FSS and primary visual cortex thinning in both groups (*r* = −0.489, *p* = 0.006; *r* = −0.389, *p* = 0.02, respectively)
Caramanos et al. (47)	110 (untreated)		Black hole lesion load (cube-rooted) in brain [3D T1w/Bayesian tissue classification]	EDSS	Significant correlation between cube root of black hole lesion load and EDSS (*r* = 0.619; *p* < 0.001)
Ramasamy et al. (48)	88		Regional subcortical tissue volume Cortical thickness [3D T1w/FreeSurfer]	EDSS	Significant correlation between EDSS and third ventricle volume (*r* = 0.415), right caudate volume (*r* = −0.371), right accumbens volume (*r* = −0.411), right parahippocampal thickness (*r* = −0.409), left lateral occipital thickness (*r* = −0.360), and left postcentral thickness (*r* = −0.421) (all *p* ≤ 0.01)
Van de Pavert et al. (49)	80		GM atrophy in the cerebellum, medial temporal lobe, postcentral gyrus, precentral gyrus, insula, prefrontal cortex and thalamus [3D T1w/SPM]	EDSS T25FW 9HPT	Voxel-wise models: No correlation with volume loss and any clinical metric Region of interest analyses: EDSS: correlated with GM volume in cerebellum (adjusted *r*^2^ = 0.203; *p* = 0.018) and postcentral gyrus (adjusted *r*^2^ = 0.242; *p* = 0.002) T25FW: correlated with GM volume in cerebellum (adjusted *r*^2^ = 0.156; *p* = 0.02) and postcentral gyrus (adjusted *r*^2^ = 0.164; *p* = 0.014) 9HPT: correlated with GM volume in cerebellum (adjusted *r*^2^ = 0.100; *p* = 0.016)
Motl et al. (50)	79		Volumes of subcortical GM structures (thalamus, caudate, putamen, and pallidum) [3D T1w/SIENAX]	T25FW	Thalamus volume partially accounted for compromised ambulation in MS patients compared with controls
Anderson et al. (51)	73		Cerebellar GM volume Cerebellar WM volume [3D T1w/SPM]	Cerebellar FSS 9HPT T25FW	Cerebellar GM volume significantly lower in those with cerebellar dysfunction vs those without (*p* = 0.001); borderline significance for cerebellar WM volume (*p* = 0.059) Significant association between 9HPT and cerebellar GM volume (but not cerebellar WM volume) in multiple regression model (*p* = 0.001) No significant association between cerebellar GM or WM volume and T25FW
Motl et al. (52)	61		Volume of subcortical GM structures (thalamus, caudate, putamen and pallidum) Global WM volume Global GM volume [3D T1w/SIENAX]	6 MW T25FW	Significant correlation between global WM volume and 6 MW and T25FW (*p* < 0.01 for both) Significant correlation between global GM volume and 6 MW (*p* < 0.05) Significant correlation between 6 MW and T25FW and volumes of the thalamus, caudate, pallidum and putamen (*p* < 0.05 for putamen; *p* < 0.01 for others) Results for caudate and pallidum remained significant after controlling for age, MS clinical course, and whole brain GM and WM volumes (*p* < 0.05) Linear regression: pallidum volume was the only significant correlate of 6 MW and T25FW performance (*p* < 0.01)
Shiee et al. (53)	60		Cortical GM volume Cerebral WM volume Cerebral volume fraction Volumes of caudate nucleus, putamen, thalamus, ventricles and brainstem [3D T1w/TOADS-CRUISE]	EDSS MSFC MSSS	EDSS (*r* = −0.40; *p* = 0.001), MSFC (*r* = 0.35; *p* = 0.005), and 9HPT (*r* = −0.45; *p* < 0.001) correlated with WM volume 9HPT and MSFC correlated with cerebral volume fraction [*r* = −0.46 (*p* < 0.001) and *r* = 0.39 (*p* = 0.001), respectively], ventricle [*r* = 0.47 (*p* < 0.001) and *r* = −0.42 (*p* = 0.001), respectively] and thalamus volumes [*r* = −0.35 (*p* = 0.005) and *r* = 0.34 (*p* = 0.007), respectively] EDSS (*r* = −0.34; *p* = 0.007) and T25FW (*r* = −0.32; *p* = 0.01) correlated with brainstem volume T25FW correlated with thalamus volume (*r* = −0.32; *p* = 0.01)
Jaworski et al. (54)	48		Brain atrophy (BPF) [T1w/Jim software]	EDSS MSSS	Brain atrophy correlated with EDSS (*r* = −0.51; *p* = 0.0002) and MSSS (*r* = −0.42; *p* = 0.002)
Thaler et al. (55)	40		Black holes [3D T1w/Lesion Segmentation Tool]	EDSS MSFC	Significant correlations between black hole volume and clinical disability (*r* = 0.333 to *r* = 0.442; *p* = 0.039 to *p* = 0.004)
Granberg et al. (56)	37		Corpus callosum area Corpus callosum index (CCI) Corpus callosum volume Brain volume GM volume WM volume [3D T1w/Freesurfer and Lesion Segmentation Toolbox]	EDSS	Significant correlations between EDSS and: Corpus callosum area (*r* = −0.56; *p* < 0.001) CCI (*r* = −0.45; *p* = 0.001) Corpus callosum volume (*r* = −0.55; *p* < 0.001) Brain volume (*r* = −0.45; *p* = 0.001) GM volume (*r* = −0.50; *p* < 0.001)
Sbardella et al. (57)	36		Regional GM volume WM volume [3D T1w/VBM]	EDSS MSFC	Significant correlation between cerebellar volume and 9HPT (*p* < 0.05)
Chu et al. (58)	26		BPV [3D T1w/SIENAX]	EDSS T25FW	1.5 T MRI: BPV correlated with EDSS (*r* = −0.43; *p* = 0.027) and T25FW (*r* = −0.46; *p* = 0.018) 3 T MRI: BPV correlated with EDSS (*r* = −0.49; *p* = 0.011) and T25FW (*r* = −0.56; *p* = 0.003)
Tam et al. (59)	24		Black hole volumes [T1w/Semi-automated method]	EDSS	Significant correlation between black hole volume and EDSS
Zimmermann et al. (60)	19 (with predominantly spinal cord lesions)		Putamen fraction Putamen volume/BPF [3D T1w/VBM and ROI-based analyses]	EDSS MSSS	Significant correlation between putamen fraction and MSSS (*r* = −0.521; *p* = 0.027)
Gorgoraptis et al. (61)	11 patients with history of hemiparesis due to corticospinal tract lesion		Volume, thickness, surface area and curvature of precentral and paracentral cortices [3D T1w/FreeSurfer]	EDSS Pyramidal FSS T25FW 9HPT	Significant correlation between: Paracentral cortex volume and T25FW (*r* = −0.71; *p* = 0.022) Paracentral cortex surface area (*r* = −0.65; *p* = 0.030) and curvature (*r* = −0.63; *p* = 0.037) and pyramidal FSS No correlation between cortical thickness and disability
**COMPARISON OF SUBTYPES**					
Varoğlu et al. (62)	RRMS (*n* = 14) and SPMS (*n* = 13)		Cerebellar volume [T2w FLAIR/Cavalieri method]	EDSS	Cerebellar volume was negatively correlated with EDSS in both groups of patients (*r* = 0.896 for RRMS, *r* = −0.854 for SPMS; *p* < 0.01 for both)
Anderson et al. (63)	RRMS (*n* = 14) and PPMS (*n* = 12)		Cerebellar GM atrophy Cerebellar WM atrophy [3D T1w/SPM]	EDSS Cerebellar FSS 9HPT T25FW	Cerebellar WM volume was associated with 9HPT in patients with PPMS, independently of cerebellar GM volume No association between cerebellar GM volume and any of the disability measurements
**(b) Longitudinal studies**					
**Reference**	**Follow-up period (years)**	**Patients (*n*)**	**MRI measure of neurodegeneration [acquisition/quantification methods]**	**Measure of disability**	**Results**
**RRMS**					
Hofstetter et al. (64)	1	239	Regional GM volume [3D T1w/VBM]	EDSS MSFC	Significant difference in volume of right precuneus (*p* < 0.001) and postcentral gyrus (*p* < 0.001) between patients with stable and progressive disability measured using EDSS
Vaneckova et al. (65)	≤5	181	Brain volume (BPF) [3D T1w/In-house software]	EDSS	Patients with low baseline lesion load: significant correlation between increased brain atrophy in first 2 years and increase in EDSS at years 4 and 5 (*r* ≤ −0.71; *p* < 0.01) Patients with high baseline lesion load: no correlation between early brain atrophy and later change in EDSS
Giorgio et al. (66)	10 (±0.5)	58	Black holes [T1w/Jim software]	EDSS	Higher EDSS at 10 years correlated with greater baseline black hole number (*r* = 0.53; *p* < 0.001) and volume (*r* = 0.42; *p* < 0.001) Moderate correlation between increase in EDSS and increasing black hole volume over 10 years (*r* = 0.47; *p* < 0.001) In stepwise multiple regression analysis, increase in EDSS over 10 years was best correlated with the combination of baseline black hole number and increasing black hole volume (*r* = 0.61; *p* < 0.001)
**RELAPSING ONSET**					
Rocca et al. (67)	8	73	Thalamic fraction [PD-weighted images/Manual segmentation]	EDSS	Baseline thalamic fraction was an independent predictor of worsening disability at 8 years (OR = 0.62; *p* = 0.01)
**PPMS**					
Mesaros et al. (68)	1.25 (mean)	54	Thalamic volume [PD-weighted images/SPM]	EDSS	Neither baseline thalamic volume nor the average change in thalamic volume were predictive of increase in EDSS in univariate analysis
Eshaghi et al. (69)	5	36	Volume of GM structures [3D T1wR/VBM]	EDSS MSFC	Higher rate of volume loss in the bilateral cingulate cortex associated with greater clinical disability (MSFC) measured at 5 years (*r* = 0.49; *p* = 0.003)
**MS**					
Tedeschi et al. (70)	2	267	Abnormal WM fraction NAWM fraction Global WM fraction GM fraction Whole brain fraction [T1w and dual echo/multispectral, fully automated method]	EDSS	Significant correlation between all MRI parameters and EDSS at end of follow-up (*p* < 0.0001); *r* = −0.423 for GM fraction, *r* = −0.431 for whole brain fraction, *r* = −0.256 for global WM fraction, *r* = −0.220 for NAWM fraction and *r* = 0.267 for abnormal WM fraction Baseline GM fraction and whole brain fraction significantly lower in patients with progression of disability vs those with stable or improved disability (*p* < 0.05) Baseline MRI measures not related to EDSS change during follow-up
Gauthier et al. (71)	≤5	218	Brain volume (BPF) [Dual echo PD and T2w/template-driven segmentation]	EDSS	Univariate analysis: lowest baseline BPF quartile was associated with EDSS progression (OR = 1.99; *p* = 0.02) Covariate specific disability curves: in patients with 6-month EDSS of 2, probability of progression to EDSS of 3 within 3 years was 0.277 for a patient with low BPF and a high T2 lesion volume vs 0.055 for a patient with high BPF and a low T2 lesion volume
Yaldizli et al. (72)	7.1 (mean)	169	CCI^b^ [T1w/picture archiving and communication system]	EDSS	CCI at diagnosis significantly correlated with EDSS at diagnosis (*r* = −0.428; *p* < 0.001) Associated with disability progression, but was not an independent predictor of long-term disability
Figueira et al. (73)	5	128	CCI^b^ [T1w/semi-automated system]	EDSS	No correlation between reduction in CCI and change in EDSS
Neema et al. (74)	4	97	Brain atrophy (BPF) [T2w dual echo/automated template-driven segmentation]	EDSS	No association between baseline BPF or % change in BPF and change in disability (stable vs progressive)
Moodie et al. (76)	3.2 ± 0.3 (mean ± SD)	84	Brain volume (BPF)^c^ [Dual echo/automated template-driven segmentation]	EDSS	No significant association between baseline BPF and EDSS-defined clinical progression
Jacobsen et al. (77)	5 and 10	81	Brain volume WM volume Regional GM volume Volume of subcortical deep GM structures [3D T1w/SIENAX and SIENA]	EDSS	5 years: significantly higher brain (*p* < 0.001), cortical (*p* = 0.009), and putamen volume changes (*p* = 0.003) in patients with disability progression vs those without progression; no significant difference in WM volume between groups 10 years: trend for greater decrease in whole brain volume (*p* = 0.015) in patients with disability progression [Level for statistical significance set at *p* < 0.01]
Filippi et al. (78)	13	73	Black holes GM fraction WM fraction Thalamic fraction [Black holes: T1w/semi-automated local thresholding technique][GM/WM fraction: T1w/SPM][Thalamic: PD-weighted images/Manual segmentation]	EDSS MSSS	Baseline GM fraction was the only significant predictor of worsening EDSS in multivariate model (OR = 0.79; *p* = 0.01) Baseline GM fraction also predicted MSSS at follow-up (*p* = 0.0005)
Fisher et al. (79)	4	70	Brain atrophy (BPF) GM fraction WM fraction [BPF: T2w FLAIR/3D segmentation algorithm][GM fraction: T1w/intensity-based and regional probability maps][WM fraction = BPF—GM fraction]	EDSS MSFC T25FW 9HPT	GM atrophy at last visit correlated with disability; correlations were greatest with the MSFC (*r* = 0.52)
Minneboo et al. (81)	12.2 (mean)	46	Black hole lesion load BPF Ventricular fraction [Black holes: semi-automated thresholding technique][BPF and ventricular fraction: T1w spin-echo]	MSSS	Univariate analyses: Black hole lesion load (baseline and change/year) and ventricular fraction (cross-sectional and change/year) were associated with MSSS (adjusted *r*^2^ = 0.07 to 0.18; *p* = 0.063 to 0.003) Multiple regression model: Final model included change in black hole lesion load only (% of explained variance in MSSS was 28–34%)
Martola et al.^d^ (82)	9 (mean)	37	Corpus callosum area^a^ [T2w/picture archiving and communication system]	EDSS MSSS	Persisting association between corpus callosum area with disability status at baseline and end of study (*p* < 0.05)
Martola et al.^d^ (83)	9.25 (mean) 7.3–10 (range)	37	Supratentorial ventricular volume [T1w/picture archiving and communication system]	EDSS MSSS	Low to moderate association between supratentorial ventricular enlargement and disability status at baseline and end of follow-up
Martola et al.^d^ (84)	9.25 (mean) 7.3–10 (range)	37	Brain volume (BPV) Supratentorial ventricular volume [T1w/semiautomatic tool]	EDSS MSSS	Supratentorial ventricular volumes were associated with disability and this association persisted during the follow-up Annual rate of volume change in third ventricle: *p* = 0.053 for EDSS (OR = 1.36) and *p* = 0.044 for MSSS (OR = 1.52) Annual rate of volume change in lateral ventricle: *p* = 0.037 (OR = 1.24) and *p* = 0.006 (OR = 1.46), respectively
**COMPARISON OF DIFFERENT SUBTYPES**					
Pichler et al. (85)	3.6 (mean)	CIS (*n* = 63) vs definite MS (*n* = 57)	Brain volume Cortical GM volume WM volume Thalamic and basal ganglia volume [3D T1w/SIENA and SIENAX]	EDSS	No association between decline in global, compartmental or regional brain volume parameters and disability Quartiles of percentage change in brain volume were associated with disability (*p* = 0.01)
Masek et al. (86)	Not specified in abstract^e^	*n* = 12; SPMS vs RRMS vs healthy controls	Brain volume (BPV) Supratentorial ventricular volume [T1w/semiautomatic tool and BPF]	EDSS	No correlation between EDSS and black holes in SPMS, but significant correlation between increase in brain atrophy and clinical status (*p* = 0.0093)

*^a^Publications based on the same cohort of patients*.

*^b^Measure of brain atrophy*.

*^c^Measured as part of the Magnetic Resonance Disease Severity Scale, a composite MRI scale combining T1-lesions, T2-lesions, and whole brain atrophy*.

*^d^Publications based on the same cohort of patients*.

*^e^Full copy of paper not available*.

*Studies within each subsection are ordered according to size of patient population*.

*6 MW, 6-minute walk; 9HPT, 9-hole peg test; BPF, brain parenchymal fraction; BPV, brain parenchymal volume; CIS, clinically isolated syndrome; EDSS, Expanded Disability Status Scale; FLAIR, fluid-attenuated inversion recovery; FSS, Functional Systems Score; GM, grey matter; MRI, magnetic resonance imaging; MS, multiple sclerosis; MSFC, Multiple Sclerosis Functional Composite; MSSS, Multiple Sclerosis Severity Score; NAWM, normal-appearing white matter; OR, odds ratio; PD, proton density; PPMS, primary progressive multiple sclerosis; ROI, regions of interest; RRMS, relapsing-remitting multiple sclerosis; SPM, statistical parametric mapping; SPMS, secondary progressive multiple sclerosis; T1w, T1-weighted; T2LV, T2 lesion volume; T2w, T2-weighted; T25FW, timed 25-foot walk; TBM, tensor-based morphometry; VBM, voxel-based morphometry; WM, white matter*.

#### Black Holes

Historically, black holes were introduced as the first MRI measure of neurodegeneration and prevention of the evolution of newly formed lesions into persistent black holes is currently being evaluated as a possible measure of neuroprotection in several treatment trials in patients with MS.

**Figure 1:**
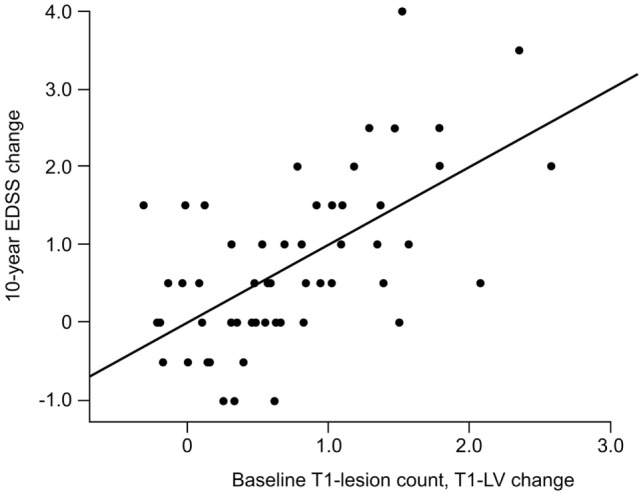
Correlation between the 10-year change in EDSS and the combined measure of baseline T1-hypointense lesion count and 10-year T1-hypointense lesion volume change (*r* = 0.61; *p* < 0.001). Reprinted from (66) by Permission of SAGE Publications, Ltd. Copyright © 2014 The authors of the original work.

The relationship between black holes and disability was assessed in seven studies (three cross-sectional and four longitudinal) (47, 55, 59, 66, 81, 86, 87). Giorgio et al. evaluated the association between black holes and EDSS scores in patients with RRMS who were followed up for 10 years (66). Higher EDSS scores at the end of the study were significantly correlated with higher numbers and volumes of black holes at baseline (*p* < 0.001 for both). Over the 10-year follow-up, there was a modest correlation between the increase in EDSS score and black hole volume (*p* < 0.001). In a stepwise multiple regression analysis, EDSS score worsening over 10 years was best associated with the combination of baseline black hole numbers and increasing black hole volume (*p* < 0.001) (Figure 1). In another study with a follow-up duration of approximately 12 years, the change in black hole lesion load was the only parameter remaining in the multiple regression model as a predictor of MSSS (81). In contrast, in the 13-year study conducted by Filippi et al., baseline black hole volume did not predict worsening disability assessed using the MSSS or EDSS (78). In a cross-sectional study of patients with untreated MS, the cube root (used to eliminate skew) of the black hole lesion load significantly correlated with the EDSS score (*p* < 0.01) (47).

Several strategies have been proposed to increase black hole detection and improve the correlation with disability, including the restriction of black hole measurements according to their intensity (59) or relaxation time thresholds (55).

#### Whole Brain Atrophy

Twenty studies (7 cross-sectional and 13 longitudinal) evaluated the association between whole brain atrophy and disability; most included patients with mixed disease phenotype. Five of the cross-sectional studies reported a significant correlation between whole brain atrophy and EDSS (*p* < 0.05 for all; see Table 1 for individual *p*-values) (37, 43, 54, 56, 58). In the sixth cross-sectional study, the level of brain atrophy was significantly greater in patients requiring ambulatory assistance compared with those not requiring assistance (*p* = 0.001) (42).

Of the 13 longitudinal studies, 5 demonstrated a correlation between brain atrophy and disability (75, 77, 80, 81, 86), while 4 indicated that there was no correlation (74, 76, 79, 84). Among these, the study by Jacobsen et al. had the longest follow-up period (5 and 10 years) (77). At 5 years, patients with disability progression had significantly greater whole brain volume loss than those with no progression (*p* < 0.001), while at 10 years, there was a trend for greater decrease in whole brain volume in patients with disability progression (*p* = 0.015; statistical significance set at *p* < 0.01) (77).

Results were mixed in two of the other longitudinal studies. In the study by Tedeschi et al., which included 267 patients with MS, there was a significant correlation between baseline brain volume and the EDSS score at follow-up (2 years) (*p* < 0.0001). In addition, brain volume was significantly lower in patients with progression vs those with stable or improved disability (*p* < 0.05). However, baseline brain volume was not related to the change in EDSS score during the follow-up period (70). Pichler et al. found that although there was no association between the decline in whole brain volume and disability, quartiles of percentage change in brain volume were associated with the degree of disability (*p* = 0.01) (85).

The two remaining longitudinal studies evaluated the predictive value of baseline brain volume and T2 lesion load for subsequent disability. In a 5-year study in patients with RRMS, Vaneckova et al. demonstrated a significant correlation between increased brain atrophy in the first 2 years and EDSS score increase at years 4 and 5 in patients with a low lesion load at baseline (*p* < 0.01); this correlation was not observed for those with a high baseline lesion burden (65). In another study, the probability of sustained disability progression (an EDSS score ≥3 within 3 years) was almost five times higher in patients with a low brain volume and a high T2 lesion volume compared with patients with a high brain volume and low T2 lesion volume (71).

#### GM Atrophy

##### Global

Twelve studies (eight cross-sectional and four longitudinal) evaluated the correlation between global GM loss and disability. Of the six cross-sectional studies that assessed disability using the EDSS, three studies [including one with a large patient population (*n* = 927)] demonstrated a significant correlation with EDSS score (26, 40, 56), while three showed no significant correlation (28, 32). In the cross-sectional study by Motl et al., GM volume significantly correlated with results of the 6-minute walk (6 MW; *p* < 0.05), but not the T25FW (52). In the remaining cross-sectional study, in patients with MS, there was a significant difference in GM atrophy in those requiring ambulatory assistance vs those who did not (*p* = 0.0008) (42).

**Figure 2:**
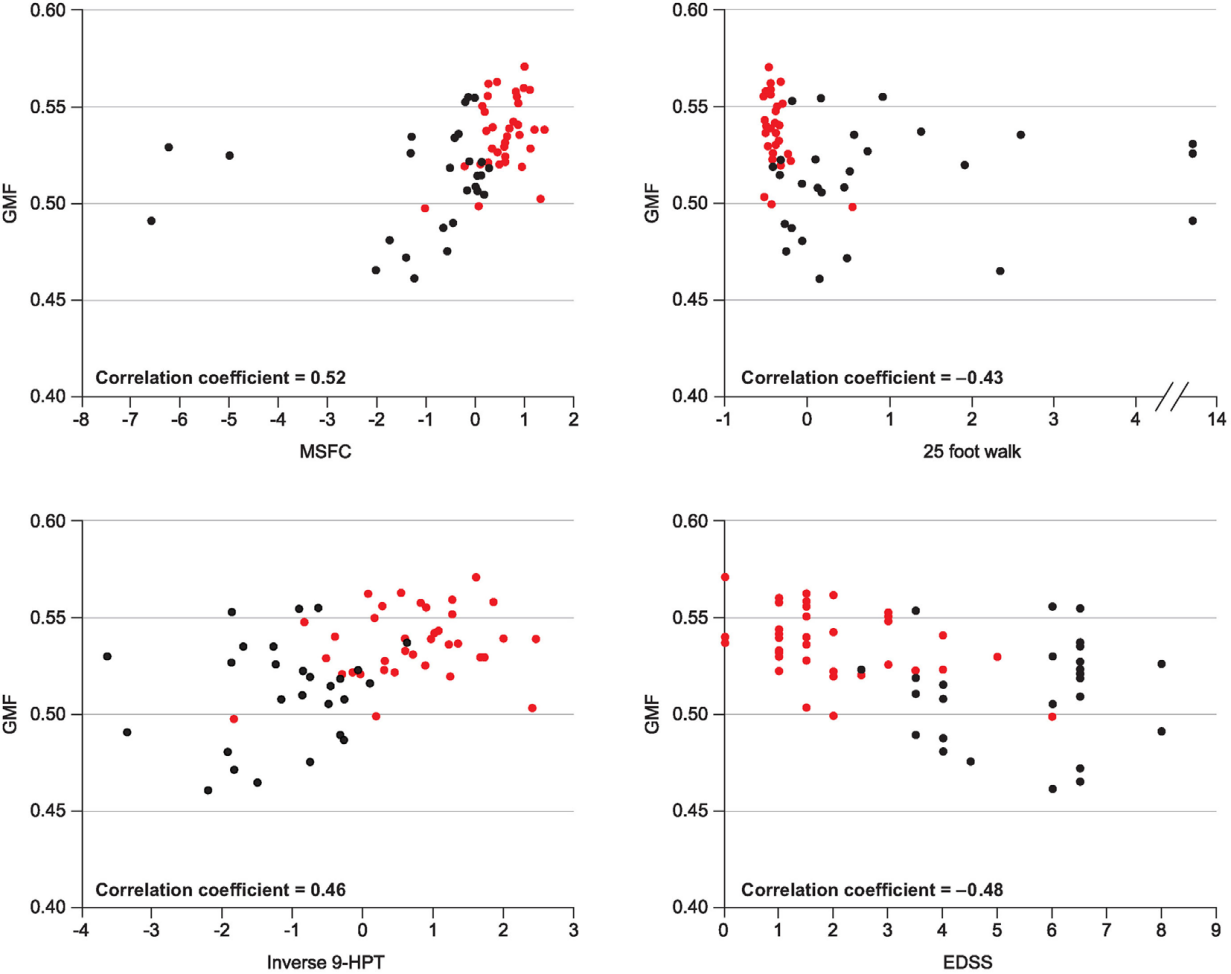
Correlation between GMF and various clinical measures of disability. Republished with permission of John Wiley and Sons Inc, from (79).

In the 13-year longitudinal study of patients with MS conducted by Filippi et al., a lower baseline GM fraction predicted worsening disability at final follow-up, as assessed using EDSS (*p* = 0.01) and MSSS (*p* = 0.0005) (78). A correlation between GM atrophy and disability (the MSFC score in particular) was also noted in a longitudinal study in an MS population that included patients with CIS, RRMS, and secondary progressive multiple sclerosis (SPMS) (Figure 2) (79). In the large 2-year longitudinal study of patients with MS (78% of whom had RRMS) conducted by Tedeschi et al., there was a significant correlation between GM volume and EDSS score at the end of the follow-up period (*p* < 0.0001), and baseline GM volume was significantly lower in patients with disability progression compared with those who did not progress (*p* < 0.05) (70). However, baseline GM atrophy was not related to EDSS change during the 2-year follow-up period. Finally, in the study by Rudick et al., a low baseline GM fraction correlated with an EDSS score ≥6 at final follow-up (mean, 6.6 years), and patients with disability progression (measured using the MSFC, but not the EDSS) had significantly higher GM atrophy rates compared with those who did not progress (*p* = 0.03) (80).

##### Regional GM, Including Specific GM Structures

Several studies have applied different methods of analysis to assess the role of atrophy of specific GM structures (cortex, deep GM structures, etc.) in disability. Many of these studies (mostly cross-sectional) have evaluated the association between cortical GM loss and disability, but with mixed results (see Table 1). The largest of the cross-sectional studies included 208 patients with RRMS, PPMS, or SPMS (41), 128 patients with RRMS (28), and 115 patients with CIS, possible MS, RRMS, or SPMS (46). In the study by Steenwijk et al., reduced cortical thickness was one of the significant predictors of EDSS in a multivariate model (*p* < 0.05) (41). In the study by Prinster et al., there was a correlation between EDSS score and GM loss in the bilateral primary motor and somatosensory areas and middle frontal gyri (28). Calabrese et al. were unable to demonstrate a correlation between diffuse cortical thinning and EDSS score; however, significant correlations were observed between some of the functional system scores and atrophy of the corresponding cortical areas, e.g., the visual functional system score and atrophy of the visual cortex (46).

Three of the four longitudinal studies demonstrated an association between disability and atrophy of the cortex (*p* = 0.009) (77), the bilateral cingulate cortex (*p* = 0.003) (69), and the right precuneus and postcentral gyrus (*p* < 0.001 for both) (64). In the fourth longitudinal study, conducted in patients with CIS or definite MS, there was no association between the decline in cortical GM volume and progression of disability (85).

A number of studies have shown that there is an association between disability and GM atrophy in the thalamus and basal ganglia (29, 35, 48, 50, 52, 53, 60, 67, 77). For example, Tao et al. demonstrated a significant correlation between atrophy of the thalamus, caudate nucleus, and putamen and EDSS score in a cross-sectional study in patients with relapsing MS (all *p* < 0.0001) (35). In another cross-sectional study in patients with RRMS, those with EDSS scores >3.0 had more severe GM atrophy in the left basal ganglia and thalamus compared with those with scores ≤3.0 (29). In the longitudinal study conducted by Jacobsen et al., atrophy of the putamen was implicated in disability progression at 5 years (77), and early thalamic atrophy was an independent predictor of disability at 8 years in the study by Rocca et al. (67). However, other studies have not shown a correlation between disability and GM loss in the thalamus and/or basal ganglia (31, 68, 78). This includes the 13-year longitudinal study by Filippi et al., in which baseline thalamic fraction was not an independent predictor of change in EDSS score or MSSS at the final follow-up (78). Also, in the study by Mesaros et al., neither baseline nor mean change in thalamic volume significantly correlated with change in EDSS score over 5 years (68).

Another structure that has been evaluated in a number of studies is the cerebellum. These studies have shown a significant correlation between GM atrophy of the cerebellum and EDSS score (27, 49) and 9HPT (49, 51, 57); the correlation with T25FW was statistically significant in one study (49), but not another (51). In the small study by Anderson et al., however, there was no significant correlation between cerebellar GM volume and a number of disability measures, including EDSS and 9HPT (63). D’Ambrosio et al. evaluated the correlation between the EDSS/9HPT and whole and sub-regional cerebellar volumes; in a multivariate analysis, only the anterior cerebellar volume remained significant (*p* = 0.003 for the EDSS and *p* = 0.02 for the 9HPT) (36).

#### WM Atrophy

Thirteen studies (seven cross-sectional and six longitudinal) assessed the association between global WM atrophy and disability (39, 40, 42, 52, 56, 57, 70, 77–80, 85). In the cross-sectional study by Prinster et al., WM loss correlated with EDSS score (*p* < 0.0001) (28), while Howard et al. demonstrated a significant difference in WM atrophy in patients requiring ambulatory assistance vs those who did not (*p* = 0.02) (42). Although Motl et al. showed that there was a significant correlation between global WM atrophy and results of the 6 MW and T25FW tests (*p* < 0.01 for both), these did not survive in linear regression analysis (52). Three cross-sectional studies—including the large study (*n* = 927) conducted by Roosendaal et al.—did not show a correlation between WM atrophy and EDSS score (40, 56).

In five of the longitudinal studies, there was no association between global WM atrophy and disability (26, 77–79, 85). In the longitudinal study by Tedeschi et al., there was a significant correlation between WM atrophy and EDSS score at the end of the follow-up period (2 years) (*p* < 0.0001), but not with the change in the EDSS score during follow-up (70). Finally, in the study by Rudick et al., a lower baseline WM fraction correlated with an EDSS score ≥6 at final follow-up (mean, 6.6 years), but the level of WM atrophy was similar in patients with and without MSFC progression (80).

Three studies (two longitudinal and one cross-sectional) evaluated the association between the corpus callosum index (CCI; a measure that is thought to reflect brain atrophy) and disability. In one longitudinal study, the CCI correlated with EDSS score at diagnosis, but did not predict 7-year disability (72). In the second longitudinal study, baseline CCI was able to distinguish RRMS from SPMS, but did not correlate with the EDSS score after 5 years (73). In the cross-sectional study, there was a significant correlation between the CCI and EDSS score (56). Studies have also evaluated CC area and volume, but with mixed results (34, 56, 82); one of these studies was longitudinal and demonstrated a persistent association between the corpus callosum area and disability during a mean follow-up of 9 years (*p* < 0.05) (82).

### Spinal Cord Atrophy

Spinal cord abnormalities at the onset of MS have important prognostic implications and extensive spinal cord pathology is common as the disease progresses (88). Sixteen studies were identified evaluating the relationship between spinal cord atrophy and disability (Table 2); all but one (89) were cross-sectional, although the study by Yiannakas et al. included a longitudinal subgroup. These studies consistently demonstrated a significant correlation between clinical disability and cervical cord cross-sectional area (CSA) at various cord levels (89–100) as well as regional (C2/C3) and overall volume of the cervical cord (101). Exceptions were the studies by Weier et al. in 202 patients with MS, which found a weak correlation between signs of spinal cord atrophy and EDSS scores (102), and the study by Blamire et al. (*n* = 11), which found no correlation between spinal cord atrophy and various measures of disability (103). The largest study, which included 335 patients with MS, demonstrated that although cord CSA correlated with EDSS in the overall population (*p* < 0.0001), there were different effects according to MS clinical phenotype. The association was significant for RRMS (*p* = 0.001), SPMS (*p* = 0.001), and PPMS (*p* = 0.01), but not for CIS or benign MS (95).

**Table 2:** MRI studies evaluating the relationship between spinal cord atrophy and disability.

Reference	Patients (*n*)	MRI measure of neurodegeneration [acquisition/quantification methods]	Measure of disability	Results
**CROSS-SECTIONAL STUDIES**				
Rocca et al. (95)	335 with MS	Cervical cord CSA (C2 to C5) [3D T1w/active surface method]	EDSS	Cord CSA correlated with EDSS in patients with RRMS (*r* = −0.30; *p* = 0.001), SPMS (*r* = −0.34; *p* = 0.001), and PPMS (*r* = −0.27; *p* = 0.01), but not in patients with CIS or benign MS
Biberacher et al. (91)	267 with CIS or RRMS	Upper cervical cord CSA at C2/C3 [3D T1w/FSL software]	EDSS	Cord CSA correlated with EDSS (*r* = −0.131; *p* = 0.044)
Weier et al. (102)	202 with MS	Whole spinal cord atrophy [T2w/visual assessment]	EDSS	Weak correlation between cord atrophy and EDSS scores (*r* = 0.30)
Daams et al. (92)	196 with MS	Upper cervical cord CSA [3D T1w/semi-automated method]	EDSS T25FW 9HPT Cord Functional Score	Cord area was independently associated with EDSS (*r* = −0.296; *p* < 0.001), T25FW (*r* = 0.240; *p* = 0.001), and 9HPT (*r* = −0.206; *p* = 0.005)
Bernitsas et al. (90)	150 with MS	Cervical cord CSA (C2) [3D T1w/Losseff semi-automated method (104)]	EDSS	Significant correlation between CSA-C2 and EDSS (*r* = −0.75; *p* < 0.0001) Multivariable regression showed that CSA-C2 was a significant predictor of disability independent of disease duration and phenotype (*p* < 0.0001)
Oh et al. (94)	133 with MS	C3–C4 cord volume [3D T1w/fully automated segmentation protocol (105)]	EDSS MSFC Hip flexion strength Vibration sensation threshold	Correlations between clinical measures (EDSS: *r* = −0.20, *p* = 0.02; MSFC: *r* = 0.16, *p* = 0.06; hip flexion strength: *r* = 0.35, *p* = 0.0001; vibration threshold: *r* = −0.19, *p* = 0.03) and cord volume
Yiannakas et al. (99)	120 with MS (40 in longitudinal subgroup; 1-year follow-up)	Cervical cord CSA (two segments: C2/C3 and C2/C5) [3D T1w/Propseg vs semi-automated active surface method]	EDSS MSFC T25FW 9HPT ASIA motor and sensory scores	Baseline CSA was significantly associated with baseline clinical variables (both segments) (*p* < 0.001 for all) CSA measures at 1 year were significantly associated with ASIA motor and sensory scores only (*p* = 0.048 to *p* = 0.001) Baseline CSA for both segments predicted ASIA motor scores at 1 year (*p* ≤ 0.003)
Schlaeger et al. (96)	113 with MS	Spinal cord WM area (C2/C3) Spinal cord GM area (C2/C3) Upper cervical cord CSA (C2/C3) [2D PSIR/Active surface method]	EDSS T25FW 9HPT	GM, WM, and cord CSA significantly correlated with EDSS (*r* = −0.60, −0.32, and −0.42, respectively; all *p* ≤ 0.001) and T25FW (*r* = −0.50, −0.28, and −0.36, respectively; *p* < 0.001, *p* = 0.004 and *p* < 0.001, respectively) GM area (*r* = −0.37) and cord CSA (*r* = −0.22) significantly correlated with 9HPT (*p* < 0.001 and *p* = 0.024, respectively) GM area was the strongest correlate of disability in multivariate models
Rocca et al. (106)	77 with MS	Regional cervical cord atrophy (voxel-based) [3D T1w/voxel-based analysis, active surface method]	EDSS FSS	SPMS: cord atrophy at C1/C2 correlated with pyramidal FSS (*r* = −0.91; *p* < 0.001) PPMS: cord atrophy at C1/C2 correlated with EDSS (*r* = −0.68) and pyramidal FSS (*r* = −0.89) (*p* < 0.001) No correlation between regional cord atrophy and clinical variables for other MS phenotypes
Valsasina et al. (98)	71 with RRMS or SPMS	Regional cervical cord atrophy [3D T1w/voxel-based analysis, active surface method]	EDSS	Regional cervical cord atrophy was correlated with clinical disability (*r* = −0.46 to −0.57; *p* < 0.001)
Benedetti et al. (100)	68 with benign MS or SPMS	Cervical cord CSA [3D T1w/semi-automated method of Losseff (104)]	EDSS	Cord CSA was an independent predictor of EDSS (*p* = 0.001)
Horsfield et al. (93)	40 with RRMS or SPMS	Cervical cord CSA (C2 and C2–C5) [3D T1w/semiautomatic active surface vs Losseff method (104)]	EDSS Ambulation index	Strong correlations between the EDSS (C2: *r* = −0.51; C2–C5: *r* = −0.59) and ambulation index (C2: *r* = −0.58; C2–C5: *r* = −0.648) and CSA (*p* < 0.001)
Healy et al. (101)	34 with MS	C2–3 volume Cervical cord volume Thoracic cord volume Whole cord volume [T2-weighted sequence/JIM software]	EDSS	C2–3 volume and cervical cord volume correlated with EDSS score (*p* < 0.05)
Song et al. (97)	29 with MS	Upper cervical cord CSA [3D T1w and T2w/semi-automated software (107)]	EDSS	Stronger correlation between EDSS and normalized measurement of cord area vs absolute measurement [*r* = −0.84 (*p* < 0.01) vs *r* = −0.46 (*p* < 0.05)]
Blamire et al. (103)	11	Spinal cord CSA (C2–C5) [T1w/Jim software]	EDSS 9PHT T25FW	No correlation between cord atrophy and measures of disability
**LONGITUDINAL STUDIES**				
Valsasina et al. (89)	35 with MS (mean follow-up, 2.3 years)	Cervical cord CSA [3D T1w/active surface method vs Losseff method]	EDSS	At baseline, there was a significant correlation between EDSS and both methods used to measure CSA (AS method: *r* = −0.59; *p* < 0.001; Losseff method: *r* = −0.40; *p* = 0.01) At follow-up, AS cord CSA (but not CSA evaluated using the Losseff method) correlated with EDSS (*r* = −0.50; *p* = 0.002)

*Studies within each subsection are ordered according to size of patient population*.

*9HPT, 9-hole peg test; AS, active surface; ASIA, American Spinal Injury Association; CIS, clinically isolated syndrome; CSA, cross-sectional area; EDSS, Expanded Disability Status Scale; FSS, Functional Scale Score; GM, grey matter; MRI, magnetic resonance imaging; MS, multiple sclerosis; MSFC, Multiple Sclerosis Functional Composite; RRMS, relapsing-remitting multiple sclerosis; SPMS, secondary progressive multiple sclerosis; T1w, T1-weighted; T2w, T2-weighted; T25FW, timed 25-foot walk; WM, white matter*.

In the longitudinal study of 35 patients with MS conducted by Valsasina et al., there were significant associations between cord CSA and EDSS, both at baseline and follow-up (89). In the subgroup analysis of 40 patients from the study conducted by Yiannakas et al. who were followed up for 1 year, cervical spinal cord CSA at the end of follow-up was significantly associated with American Spinal Injury Association (ASIA) motor and sensory scores (*p* = 0.048 to *p* = 0.001), but not with EDSS, MSFC, T25FW, or 9HPT (99). Cord CSA predicted ASIA motor scores at 1 year (*p* ≤ 0.001) (99).

The association between regional cervical cord involvement and disability has also been explored. In the voxel-based study by Valsasina et al., regional cord atrophy was more widespread in patients with SPMS than in those with RRMS. In the overall population, cervical cord atrophy correlated with clinical disability (*p* < 0.001) (98). In the study by Rocca et al., the regional distribution of cord atrophy differed significantly among the main MS clinical phenotypes. Regional cord atrophy was correlated with clinical disability and impairment in the pyramidal system for progressive MS (*p* < 0.001), but there was no correlation between cord atrophy and disability for the other MS phenotypes (CIS, RRMS, and benign MS) (106).

Schlaeger et al. evaluated the association between spinal cord WM and GM area and various measures of disability (96). They demonstrated that GM and WM area (as well as CSA) correlated significantly with EDSS score (*p* ≤ 0.001 for both) and T25FW results (*p* < 0.001 and *p* = 0.004, respectively), whereas only the GM area correlated significantly with the 9HPT results (*p* = 0.024). In a multivariate model, spinal cord GM area was the strongest correlate of the EDSS score (96). In another study by the same group, which evaluated both brain and spinal cord atrophy (see next section), there was a significant correlation between thoracic cord GM area and lower limb function (108).

### Brain and Spinal Cord Atrophy

Fifteen studies (mainly cross-sectional) have evaluated both brain and spinal atrophy correlation with disability (Table 3). Two of the largest studies, one cross-sectional and one longitudinal, were conducted by Lukas et al. (109, 110). In the cross-sectional study, which included 440 patients with MS, spinal cord (but not brain) atrophy and brain black hole volume were independent explanatory factors for the EDSS score, while spinal cord and GM brain atrophy were the strongest explanatory factors for physical disability measured using the T25FW (110). In the longitudinal study, in which 352 patients with MS were followed up for 2 years, baseline cord CSA (*p* = 0.03) and the annualized percentage change in brain volume (*p* = 0.07) were significant predictors of disability progression (EDSS score change) at year 2 (109).

**Table 3:** MRI studies evaluating the relationship between brain and spinal cord black holes/atrophy and disability.

(a) Cross-sectional studies					
Reference	Patients (*n*)	MRI measure of neurodegeneration [acquisition/quantification methods]		Measure of disability	Results
**CIS**					
Bonati et al. (111)	70 (patients were assessed 20 years after presentation with CIS)	Upper cervical cord CSA GM fraction [Cord CSA: 3D T1w/semi-automated method of Losseff et al (104)][GM fraction: 3D T1w/SPM]		EDSS MSFC 9HPT T25FW	Cord CSA Significant correlation with EDSS (*r* = −0.42; *p* < 0.001), MSFC (*r* = 0.42; *p* < 0.001), 9HPT (*r* = 0.39; *p* = 0.001), T25FW (*r* = −0.34; *p* = 0.004) GM fraction Significant correlation with EDSS (*r* = −0.47; *p* < 0.001), MSFC (*r* = 0.56; *p* < 0.001), 9HPT (*r* = 0.60; *p* < 0.001), T25FW (*r* = −0.42; *p* = 0.001) Cord CSA and GM fraction were independently associated with EDSS and MSFC
**PPMS**					
Ruggieri et al. (112)	26	Brain volume Deep GM volume Cervical cord CSA Cervical cord volume [3D T1w/SIENAX (brain volume) and active surface method (spinal cord)]		EDSS T25FW 9HPT	Significant correlation between 9HPT results (non-dominant hand) and thalamic volume (*r* = −0.48; *p* = 0.02) and spinal cord volume (*r* = −0.44; *p* = 0.03) No association between brain and WM volumes and 9HPT for non-dominant hand
Kolind et al. (113)	15 (PPMS)	Brain volume (ventricular cerebrospinal fluid) Cervical cord volume [3D T1w/SIENAX (brain volume) and semiautomatic method (114) (cord volume)]		EDSS MSFC 9HPT T25FW	Brain volume correlated with MSFC (*r* = −0.73; *p* = 0.002), 9HPT (*r* = −0.67; *p* = 0.007), but not MSFC or T25FW Cervical cord volume correlated with T25FW only (*r* = −0.54; *p* = 0.04)
**SPMS**					
Furby et al. (115)	117	Brain volume GM volume WM volume Central cerebral volume Cervical cord CSA (C2/C3) [Brain/GM/WM volume: 3D T1w/SIENAX][Central cerebral volume: 2D T1w/Losseff et al. (116)][Cord CSA: 3D T1w/Losseff et al. (104)]		EDSS MSFC	All MRI measures correlated significantly with MSFC; strongest correlation with brain volume (*r* = 0.47; *p* < 0.001) Stepwise regression model: Only brain volume (*p* = 0.001) and cervical cord CSA (*p* = 0.008) were significant independent predictors of MSFC Cervical cord CSA was the only measure with significant association with EDSS score (*r* = −0.22; *p* = 0.02)
**MS**					
Lukas et al. (110)	440	Upper cervical cord CSA Brain black holes Brain volume GM volume WM volume [Brain/GM/WM volume: 3D T1w/SEINAX][Black holes: 3D T1w/AMIRA semiautomatic software][Cord CSA: 3D T1w/semi-automated segmentation method]		EDSS T25FW 9HPT	Cord CSA correlated with EDSS score (*r* = −0.39) and T25FW and 9HPT (*r* ≤ −0.27) (*p* < 0.001 for all comparisons) Cord CSA and number of brain black holes were the strongest explanatory factors for EDSS score Cord CSA and GM volume were the strongest explanatory factors for T25FW
Kearney et al. (117)	159	Brain GM fraction Brain WM fraction Upper cervical spinal cord CSA [3D T1w/SPM (GM and WM fraction) and active surface method (cord CSA)]		EDSS	Significant correlation between EDSS and WM fraction (*r* = −0.32; *p* < 0.01) and cord CSA (*r* = −0.31; *p* < 0.01) Binary model: cord CSA associated with requirement for walking aid (EDSS score ≥ 6) (*p* < 0.01) 4-category EDSS model: cord CSA (*p* < 0.01) and GM fraction (*p* = 0.04) associated with disability
Schlaeger et al. (108)	142	Total cord CSA GM and WM area at disc levels, C2/C3, C3/C4, T8/9 and T9/10 Brain GM volume [Cord: 2D PSIR/Active surface method][Brain: MP-RAGE/FreeSurfer]		EDSS T25FW 9HPT Hip flexion strength	All spinal cord measurements (GM, WM and total cord areas) correlated with EDSS score (all *p* ≤ 0.001) and T25FW (all *p* < 0.001) Thoracic cord GM areas correlated with lower limb function Multivariable model: cervical cord GM areas had strongest correlation with EDSS followed by thoracic cord GM area and brain GM volume
Oh et al. (118)	102	Cervical spinal cord CSA BPF [Cord: gradient-echo images/Automated method][Brain: diffusion tensor images/BPF]		EDSS MSFC Hip flexion strength Vibration sensation threshold	Cord CSA was an independent predictor of EDSS (beta coefficient, −0.075; *p* < 0.01), MSFC (beta coefficient, 0.013; *p* < 0.01), hip flexion strength (beta coefficient, 0.67; *p* < 0.01) and vibration threshold (beta coefficient, −0.65; *p* = 0.01) BPF was an independent predictor of MSFC (beta coefficient, 4.97; *p* < 0.01)
Kearney et al. (119)	92	Upper cervical cord area BPV [Cord: 3D-PSIR/active surface method][Brain: 3D T1w/SPM]		EDSS MSFC	Multiple regression model: Cord area was independently associated with EDSS (*p* = 0.003) BPV independently associated with 9HPT (*p* = 0.007)
Zivadinov et al. (120)	66	Cervical cord absolute volume Cervical cord fraction Cervical cord to intracranial volume fraction Brain volume (BPF) [Cord: 3D T1w/three different methods][Brain: 3D T1w/SIENAX]		EDSS	Cervical cord absolute volume (*r* = −0.51; *p* < 0.0001) and BPF (*r* = −0.43; *p* = 0.001) showed robust correlation with disability; cervical cord fraction showed modest correlation (*r* = −0.31; *p* = 0.018) Only 8% of the variance in disability was explained by brain MRI measures when co-adjusted for the amount of cervical cord atrophy
Liptak et al. (121)	45	Medulla oblongata volume Upper cervical cord volume Brain volume (BPF) [Medulla and cord: T2w/manual segmentation][Brain: dual echo spin-echo/template-driven segmentation]		EDSS Ambulation index	A model including both medulla oblongata volume and BPF better predicted ambulatory index than BPF alone (*p* = 0.04)
Liu et al. (122)	35	Upper cervical cord CSA Brain volume (BPF) GM fraction WM fraction [Cord: T2w and 3D T1w/NeuroQLab][Brain: 3D T1w/SPM]		EDSS	Cord CSA was the only independent predictor of EDSS (*r*^2^ = 0.17; *p* = 0.013)
Cohen et al. (123)	21	Brain GM volume Brain WM volume Cervical cord volume [Brain: 3D sequence/Jim software][Cord: T2w sequence/Jim software]		EDSS T25FW	Only upper cervical cord volume significantly correlated with EDSS (*r* = −0.515; *p* = 0.020); this was largely driven by the results from patients with SPMS None of the MRI variables significantly correlated with T25FW
**(b) Longitudinal**					
**Reference**	**Follow-up period (years)**	**Patients (*n***)	**MRI measure of neurodegeneration [acquisition/quantification methods]**	**Measure of disability**	**Results**
Lukas et al. (109)	1 and 2	352 with MS	Brain volume GM volume WM volume Percentage brain volume change Upper cervical cord CSA Percentage change in cervical cord CSA [Brain: 3D T1w/SIENA and SIENAX][Cord: PD and T2w/semi-automated volumetry method (110)]	EDSS	Multivariate analysis: atrophy parameters that correlated with EDSS at Year 2 were GM volume (beta coefficient, −0.003; *p* = 0.002), baseline cord CSA (beta coefficient, −0.01; *p* = 0.047) and cord atrophy rate (beta coefficient, −0.06; *p* = 0.02) over 2 years Rate of cord atrophy but not brain atrophy was significantly higher in patients with disability progression vs those with no progression (*p* = 0.003) Multivariate binary regression: significant associations between disability progression over 2 years and baseline cord CSA (*p* = 0.03) and annualized change in brain volume (*p* = 0.07) over 2 years
Furby et al. (124)	2	56 with SPMS	Whole brain volume change GM volume WM volume Central brain volume Upper cervical cord CSA [3D T1w/whole brain: SIENA][GM and WM volume: 3D T1w/SPM][Central brain volume: 2D T1w/MIDAS][Cord CSA: 3D T1w/in-house software]	EDSS MSFC 9HPT T25FW	Rates of whole brain (*r* = 0.35; *p* = 0.009), GM (*r* = 0.42; *p* = 0.002) and spinal cord atrophy (*r* = 0.34; *p* = 0.01) all correlated with change in MSFC Rate of GM atrophy was the only correlate of change in 9HPT (*r* = 0.31; *p* = 0.02) Rate of whole brain atrophy was the only correlate of change in T25FW (*r* = 0.39; *p* = 0.003)
Agosta et al. (125)	2.4 (mean)	42	Cervical cord CSA Percentage change in brain volume [Brain: T1w/SIENA][Cord: 3D T1w/method used by Losseff (104)]	EDSS	Significant correlation between baseline EDSS and cervical cord CSA (*r* = −0.39; *p* = 0.01) Baseline cord CSA correlated with increase in disability at follow-up (*r* = −0.40; *p* = 0.01)

*Studies in each section are according to size of patient population*.

*9HPT, 9-hole peg test; BPF, brain parenchymal fraction; BPV, brain parenchymal volume; CIS, clinically isolated syndrome; CSA, cross-sectional area; EDSS, Expanded Disability Status Scale; GM, grey matter; MRI, magnetic resonance imaging; MS, multiple sclerosis; MSFC, Multiple Sclerosis Functional Composite; PPMS, primary progressive multiple sclerosis; PSIR, phase-sensitive inversion recovery; SPM, statistical parametric mapping; SPMS, secondary progressive multiple sclerosis; T1w, T1-weighted; T2w, T2-weighted; T25FW, timed 25-foot walk; WM, white matter*.

In three other studies (all cross-sectional), which used multivariate regression to analyse the data, cervical cord CSA was an independent predictor of disability (118, 119, 122). In their cross-sectional study of 142 patients with MS, Schlaeger et al. used multivariate analysis to evaluate the impact on disability of various brain and spinal cord measures of atrophy (108). They found that cervical cord GM area had strongest correlation with the EDSS score, followed by thoracic cord GM area and brain GM volume.

## Discussion

This review summarizes the results of studies that have assessed the association between MRI measures of CNS neurodegeneration derived from the assessment of T1-weighted images (mostly atrophy) and disability progression in MS. Relevant studies were identified *via* a systematic evaluation of the published literature using PubMed, and it is acknowledged that some relevant studies may not have been identified if terms for atrophy and disability were not included in the publication abstract (e.g., studies that evaluated cognition as the primary endpoint). Nevertheless, over 90 studies were identified. Most, though not all, of the studies identified have shown a significant correlation between atrophy and disability. Of the various measures used to assess neurodegeneration, the most consistent results were obtained with GM and spinal cord atrophy. The results for global WM atrophy in the brain were least consistent. Overall, 18 studies assessed both global GM and global WM atrophy; in eight of these, GM but not WM atrophy was shown to correlate with disability (26, 40, 56, 78, 79, 109, 110, 124), compared with only one study showing the opposite (28). In the large study by Tedeschi et al., the EDSS at the end of the 2-year follow-up was significantly correlated with both global GM and global WM atrophy, but the correlation was stronger for the GM (*r* = −0.423 vs −0.256 for WM) (70). It is possible that degeneration of specific WM tracts contributes to disability and that a global measurement is not sensitive enough to detect this. It should also be borne in mind that conventional MRI techniques are not able to characterise and quantify all of the heterogeneous features of MS pathology (126). Several advanced MRI techniques specific to different aspects of MS pathology have been developed to evaluate the extent and distribution of microstructural tissue abnormalities in MS. Their application is contributing to improvements in the understanding of the mechanisms responsible for the presence and worsening of clinical disability. These include magnetization transfer MRI (127), which measures microstructural tissue abnormalities, and diffusion tensor imaging (128), which allows axonal and myelin injury to be quantified. In addition, the combination of postmortem MRI and histopathological evaluation is providing important insights into the abnormalities observed on MRI, enabling translation of basic pathology to the clinical setting and validation of new MRI techniques (4).

It has been suggested that combining MRI markers may increase sensitivity to disability changes. One composite that combines three MRI measures of MS severity is the Magnetic Resonance Disease Severity Scale (MRDSS), which generates a score between 0 and 10 based on T2 lesion volume, brain volume [brain parenchymal fraction (BPF)] and the ratio of the T1:T2 lesion volume (129). Although the MRDSS showed a larger effect size than any of the individual components in distinguishing patients with RRMS from those with SPMS, the correlation with the EDSS score was similar to that observed with BPF (129). In a subsequent longitudinal study, prediction of disability (EDSS score) progression was significant for T2 lesion volume only (76). More recently, the MRDSS (MRDSS2) has been revised, replacing BPF with GM fraction and adding upper spinal cord CSA (130). The correlation between MRDSS2 and EDSS score was shown to be significant in 55 patients with MS (130). Pardini et al. have proposed a composite MRI-based measure that assesses motor network integrity (131). It is based on fractional anisotropy, magnetization transfer ratio, and normalized tract volume of motor network connections. The ability of this composite measure to predict disability was substantially greater than conventional non-network-based MRI measures (131). Another approach to improving visualization of MS-induced neurodegeneration is the use of multimodal MRI acquisition (132). A correlation with disability was observed when this method was applied to cortical GM and corpus callosum WM in patients with RRMS (132).

In most of the studies in which it was evaluated, there was a correlation between black holes and disability outcome measures. This supports the concept that focal, irreversible tissue loss, as well as more diffuse loss of tissue, has an impact on disability in MS. A number of the studies assessing black holes also evaluated T2 lesion load, which represents focal WM lesions. In the largest of these studies (110), conducted in 440 patients, there was a significant correlation between T2 lesion load and EDSS in the univariate, but not the multivariate analysis, while black hole lesion load was significant in both analyses. Notably, neither parameter was significant in the multivariate analysis of the association with T25FW results (110). In the study by Caramanos et al., the correlation with EDSS was greater for black holes than T2 lesions (47), and in the study by Giorgio et al., EDSS worsening over 10 years was best correlated with the combination of baseline black hole lesion count and increasing black hole lesion volume (66).

When considering the results of the studies included in this review, it should be borne in mind that correlation does not prove causality, and multivariate analysis, to control for confounding variables, was not applied in all studies. It is also clear from the information presented (Tables 1–3) that studies evaluating atrophy and disability are relatively heterogeneous in terms of sample size and follow-up duration, and even within studies, patient populations are heterogeneous, including a range of MS phenotypes. Heterogeneity of patient populations may be of considerable significance as the study conducted by Lukas et al., which was large enough to allow comparisons between MS subtypes, demonstrated differences in spinal cord atrophy between the progressive and relapsing forms, and showed that brain GM atrophy also differed between subtypes (109). Furthermore, in the study by Rocca et al., the correlation between spinal cord atrophy and disability was significant in some MS phenotypes, but not others (95). These differences warrant further research. Another potential confounding factor is possible variations in the use of disease-modifying therapies, which are known to affect brain volume (133, 134). The most commonly used measure of disability in the studies surveyed was the EDSS score. Although this is a very well-established measure of disability, its limitations—in particular its focus on mobility and lack of sensitivity to change—are recognized (135).

Establishing a definitive link between MRI measures of neurodegeneration and disability progression would allow such measures to be used as objective surrogate markers of disease progression, with the potential to predict future disability. They could also be used to evaluate response to treatment, which will become increasingly important as research becomes more focused on developing treatments for progressive stages/forms of MS.

Brain atrophy is already being used as an outcome measure in clinical trials of disease-modifying therapies for MS. Indeed, in a meta-analysis of data from 13 trials (including >13,500 patients with RRMS), treatment effects on disability progression were correlated with treatment effects both on brain atrophy and on active MRI lesions (134). At a recent expert panel meeting, a group of MS neurologists and neuro-radiologists reviewed the current literature on brain atrophy and discussed the challenges in assessing and implementing brain atrophy measurements in clinical practice (136). Brain volume loss was considered a useful longitudinal measure of disease progression and cognitive function in patients with MS (136). However, at present, methodological constraints (e.g., standardization of acquisition, lack of robust post-processing procedures) and physiological confounding factors (e.g., degree of hydration, other medical conditions) mean that brain atrophy measurement, although sufficiently precise for cohort studies, is not suitable for confidently predicting changes in individual patients (19). It has been suggested that the CCI may be a more practical measure of neurodegeneration in MS. It has been shown to correlate with the BPF (an accepted measure of brain atrophy) and is reliable and simple to apply, without the need for sophisticated software (72, 73). However, although it was significantly correlated with disability in a cross-sectional study (56), it was not an independent predictor of long-term disability in a longitudinal study (72).

## Concluding Remarks

The evaluation of MRI measures of neurodegeneration as predictive markers of disability in MS is a highly active area of research. Over the last 10 years, a large number of MRI studies have added to the existing literature on this subject, confirming that in general, MRI measures of atrophy correlate with disability. These efforts have culminated in the use of brain atrophy measurements to assess the effects of disease-modifying therapies. Although currently limited to clinical trials, discussions are beginning on how brain atrophy measurement can be applied in clinical practice. The next 10 years promises to be equally fruitful; as MRI techniques evolve, so the pathological substrates of disability will become more clearly delineated. As we work toward this goal, we should not lose sight of the challenges, both technological and financial, of introducing these techniques into everyday clinical practice.

## Author Contributions

All the authors were responsible for defining the scope and content of the article. All the authors reviewed each draft and provided approval of the final version.

## Conflict of Interest Statement

MR has received speaker honoraria from Biogen Idec, Novartis, Teva Neurosciences, and Genzyme and receives research support from the Italian Ministry of Health and Fondazione Italiana Sclerosi Multipla. GC has received compensation for consulting services for Novartis, Teva, Sanofi, Genzyme, Merck, Biogen, Excemed, Roche, Almirall, Chugai, Receptos and Forward Pharma, and compensation for speaking activities for Novartis, Teva, Sanofi, Genzyme, Merck, Biogen, Excemed, and Roche. MF is Editor-in-Chief of the Journal of Neurology; serves on a scientific advisory board for Teva Pharmaceutical Industries; has received compensation for consulting services and/or speaking activities from Biogen Idec, Merk-Serono, Novartis, and Teva Pharmaceutical Industries; and receives research support from Biogen Idec, Teva Pharmaceutical Industries, Novartis, Italian Ministry of Health, Fondazione Italiana Sclerosi Multipla, Cure PSP, Alzheimer’s Drug Discovery Foundation (ADDF), the Jacques and Gloria Gossweiler Foundation (Switzerland), and ARiSLA (Fondazione Italiana di Ricerca per la SLA).

## References

[1] Meuth SG Bittner S Wiendl H Immunotherapy of multiple sclerosis. Acta Neuropsychiatr (2009) 21(Suppl 2):27–34. 10.1017/S092427080003266X 25384864

[2] Trip SA Miller DH Imaging in multiple sclerosis. J Neurol Neurosurg Psychiatry (2005) 76(Suppl 3):iii11–8. 10.1136/jnnp.2005.073213 16107385 PMC1765701

[3] Filippi M Rocca MA Rovaris M Clinical trials and clinical practice in multiple sclerosis: conventional and emerging magnetic resonance imaging technologies. Curr Neurol Neurosci Rep (2002) 2(3):267–76. 10.1007/s11910-002-0086-2 11937006

[4] Seewann A Kooi EJ Roosendaal SD Barkhof F van der Valk P Geurts JJ Translating pathology in multiple sclerosis: the combination of postmortem imaging, histopathology and clinical findings. Acta Neurol Scand (2009) 119(6):349–55. 10.1111/j.1600-0404.2008.01137.x 19254283

[5] Centonze D Muzio L Rossi S Furlan R Bernardi G Martino G. The link between inflammation, synaptic transmission and neurodegeneration in multiple sclerosis. Cell Death Differ (2010) 17(7):1083–91. 10.1038/cdd.2009.179 19927157

[6] Ziemann U Wahl M Hattingen E Tumani H Development of biomarkers for multiple sclerosis as a neurodegenerative disorder. Prog Neurobiol (2011) 95(4):670–85. 10.1016/j.pneurobio.2011.04.007 21524682

[7] Friese MA Schattling B Fugger L Mechanisms of neurodegeneration and axonal dysfunction in multiple sclerosis. Nat Rev Neurol (2014) 10(4):225–38. 10.1038/nrneurol.2014.37 24638138

[8] Inglese M Petracca M Imaging multiple sclerosis and other neurodegenerative diseases. Prion (2013) 7(1):47–54. 10.4161/pri.22650 23117868 PMC3609050

[9] Lassmann H van Horssen J The molecular basis of neurodegeneration in multiple sclerosis. FEBS Lett (2011) 585(23):3715–23. 10.1016/j.febslet.2011.08.004 21854776

[10] Confavreux C Vukusic S Accumulation of irreversible disability in multiple sclerosis: from epidemiology to treatment. Clin Neurol Neurosurg (2006) 108(3):327–32. 10.1016/j.clineuro.2005.11.018 16413961

[11] Lee JY Taghian K Petratos S Axonal degeneration in multiple sclerosis: can we predict and prevent permanent disability? Acta Neuropathol Commun (2014) 2:97 10.1186/s40478-014-0097-7 25159125 PMC4243718

[12] Luessi F Siffrin V Zipp F Neurodegeneration in multiple sclerosis: novel treatment strategies. Expert Rev Neurother (2012) 12(9):1061–76; quiz 1077. 10.1586/ern.12.59 23039386

[13] Nataf S. Neuroinflammation responses and neurodegeneration in multiple sclerosis. Rev Neurol (2009) 165(12):1023–8. 10.1016/j.neurol.2009.09.012 19906389

[14] van Waesberghe JH Kamphorst W De Groot CJ van Walderveen MA Castelijns JA Ravid R Axonal loss in multiple sclerosis lesions: magnetic resonance imaging insights into substrates of disability. Ann Neurol (1999) 46(5):747–54. 10.1002/1531-8249(199911)46:5<747::AID-ANA10>3.0.CO;2-4 10553992

[15] Filippi M Rocca MA Barkhof F Bruck W Chen JT Comi G Association between pathological and MRI findings in multiple sclerosis. Lancet Neurol (2012) 11(4):349–60. 10.1016/S1474-4422(12)70003-0 22441196

[16] Popescu V Klaver R Voorn P Galis-de Graaf Y Knol DL Twisk JW What drives MRI-measured cortical atrophy in multiple sclerosis? Mult Scler (2015) 21(10):1280–90. 10.1177/1352458514562440 25583833

[17] Miller DH Barkhof F Frank JA Parker GJ Thompson AJ. Measurement of atrophy in multiple sclerosis: pathological basis, methodological aspects and clinical relevance. Brain (2002) 125(Pt 8):1676–95. 10.1093/brain/awf177 12135961

[18] Radu EW Bendfeldt K Mueller-Lenke N Magon S Sprenger T Brain atrophy: an in-vivo measure of disease activity in multiple sclerosis. Swiss Med Wkly (2013) 143:w13887 10.4414/smw.2013.13887 24264439

[19] Rocca MA Battaglini M Benedict RH De Stefano N Geurts JJ Henry RG Brain MRI atrophy quantification in MS: from methods to clinical application. Neurology (2017) 88(4):403–13. 10.1212/WNL.0000000000003542 27986875 PMC5272969

[20] Rojas JI Patrucco L Miguez J Cristiano E. Brain atrophy in multiple sclerosis: therapeutic, cognitive and clinical impact. Arq Neuropsiquiatr (2016) 74(3):235–43. 10.1590/0004-282X20160015 27050854

[21] Kurtzke JF On the origin of EDSS. Mult Scler Relat Disord (2015) 4(2):95–103. 10.1016/j.msard.2015.02.003 25787185

[22] Motl RW Cohen JA Benedict R Phillips G LaRocca N Hudson LD Validity of the timed 25-foot walk as an ambulatory performance outcome measure for multiple sclerosis. Mult Scler (2017) 23(5):704–10. 10.1177/1352458517690823 28206828 PMC5405807

[23] Feys P Lamers I Francis G Benedict R Phillips G LaRocca N The nine-hole peg test as a manual dexterity performance measure for multiple sclerosis. Mult Scler (2017) 23(5):711–20. 10.1177/1352458517690824 28206826 PMC5405844

[24] Ontaneda D LaRocca N Coetzee T Rudick R NMSS MSFC Task Force. Revisiting the Multiple Sclerosis Functional Composite: proceedings from the National Multiple Sclerosis Society (NMSS) Task Force on Clinical Disability Measures. Mult Scler (2012) 18(8):1074–80. 10.1177/1352458512451512 22740488

[25] Vollmer T Huynh L Kelley C Galebach P Signorovitch J DiBernardo A Relationship between brain volume loss and cognitive outcomes among patients with multiple sclerosis: a systematic literature review. Neurol Sci (2016) 37(2):165–79. 10.1007/s10072-015-2400-1 26537494

[26] Fisniku LK Chard DT Jackson JS Anderson VM Altmann DR Miszkiel KA Gray matter atrophy is related to long-term disability in multiple sclerosis. Ann Neurol (2008) 64(3):247–54. 10.1002/ana.21423 18570297

[27] Audoin B Zaaraoui W Reuter F Rico A Malikova I Confort-Gouny S Atrophy mainly affects the limbic system and the deep grey matter at the first stage of multiple sclerosis. J Neurol Neurosurg Psychiatry (2010) 81(6):690–5. 10.1136/jnnp.2009.188748 20392976

[28] Prinster A Quarantelli M Lanzillo R Orefice G Vacca G Carotenuto B A voxel-based morphometry study of disease severity correlates in relapsing-remitting multiple sclerosis. Mult Scler (2010) 16(1):45–54. 10.1177/1352458509351896 20028706 PMC2841518

[29] Riccitelli G Rocca MA Pagani E Martinelli V Radaelli M Falini A Mapping regional grey and white matter atrophy in relapsing-remitting multiple sclerosis. Mult Scler (2012) 18(7):1027–37. 10.1177/1352458512439239 22422807

[30] Nygaard GO Walhovd KB Sowa P Chepkoech JL Bjornerud A Due-Tonnessen P Cortical thickness and surface area relate to specific symptoms in early relapsing-remitting multiple sclerosis. Mult Scler (2015) 21(4):402–14. 10.1177/1352458514543811 25139946

[31] Hasan KM Walimuni IS Abid H Datta S Wolinsky JS Narayana PA. Human brain atlas-based multimodal MRI analysis of volumetry, diffusimetry, relaxometry and lesion distribution in multiple sclerosis patients and healthy adult controls: implications for understanding the pathogenesis of multiple sclerosis and consolidation of quantitative MRI results in MS. J Neurol Sci (2012) 313(1–2):99–109. 10.1016/j.jns.2011.09.015 21978603 PMC3254796

[32] Duan Y Liu Y Liang P Jia X Yu C Qin W Comparison of grey matter atrophy between patients with neuromyelitis optica and multiple sclerosis: a voxel-based morphometry study. Eur J Radiol (2012) 81(2):e110–4. 10.1016/j.ejrad.2011.01.065 21316170

[33] Mesaros S Rocca MA Absinta M Ghezzi A Milani N Moiola L Evidence of thalamic gray matter loss in pediatric multiple sclerosis. Neurology (2008) 70(13 Pt 2):1107–12. 10.1212/01.wnl.0000291010.54692.85 18272867

[34] Llufriu S Blanco Y Martinez-Heras E Casanova-Molla J Gabilondo I Sepulveda M Influence of corpus callosum damage on cognition and physical disability in multiple sclerosis: a multimodal study. PLoS One (2012) 7(5):e37167 10.1371/journal.pone.0037167 22606347 PMC3351399

[35] Tao G Datta S He R Nelson F Wolinsky JS Narayana PA Deep gray matter atrophy in multiple sclerosis: a tensor based morphometry. J Neurol Sci (2009) 282(1–2):39–46. 10.1016/j.jns.2008.12.035 19168189 PMC2744867

[36] D’Ambrosio A Pagani E Riccitelli GC Colombo B Rodegher M Falini A Cerebellar contribution to motor and cognitive performance in multiple sclerosis: an MRI sub-regional volumetric analysis. Mult Scler (2017) 23(9):1194–203. 10.1177/1352458516674567 27760859

[37] Mineev KK Prakhova LN Il’ves AG Kataeva GV Petrov AM Reznikova TN Characteristics of neurological and cognitive status in patients with multiple sclerosis in relation to the location and volumes of demyelination foci and the severity of brain atrophy. Neurosci Behav Physiol (2009) 39(1):35–8. 10.1007/s11055-008-9086-2 19089639

[38] Bodini B Khaleeli Z Cercignani M Miller DH Thompson AJ Ciccarelli O Exploring the relationship between white matter and gray matter damage in early primary progressive multiple sclerosis: an in vivo study with TBSS and VBM. Hum Brain Mapp (2009) 30(9):2852–61. 10.1002/hbm.20713 19172648 PMC6871131

[39] Galego O Gouveia A Batista S Moura C Machado E Brain atrophy and physical disability in primary progressive multiple sclerosis: a volumetric study. Neuroradiol J (2015) 28(3):354–8. 10.1177/1971400915594984 26246109 PMC4757290

[40] Roosendaal SD Bendfeldt K Vrenken H Polman CH Borgwardt S Radue EW Grey matter volume in a large cohort of MS patients: relation to MRI parameters and disability. Mult Scler (2011) 17(9):1098–106. 10.1177/1352458511404916 21586487

[41] Steenwijk MD Geurts JJ Daams M Tijms BM Wink AM Balk LJ Cortical atrophy patterns in multiple sclerosis are non-random and clinically relevant. Brain (2016) 139(Pt 1):115–26. 10.1093/brain/awv337 26637488

[42] Howard J Battaglini M Babb JS Arienzo D Holst B Omari M MRI correlates of disability in African-Americans with multiple sclerosis. PLoS One (2012) 7(8):e43061 10.1371/journal.pone.0043061 22900088 PMC3416750

[43] Tauhid S Neema M Healy BC Weiner HL Bakshi R MRI phenotypes based on cerebral lesions and atrophy in patients with multiple sclerosis. J Neurol Sci (2014) 346(1–2):250–4. 10.1016/j.jns.2014.08.047 25220114

[44] Preziosa P Rocca MA Mesaros S Pagani E Drulovic J Stosic-Opincal T Relationship between damage to the cerebellar peduncles and clinical disability in multiple sclerosis. Radiology (2014) 271(3):822–30. 10.1148/radiol.13132142 24555637

[45] Yaldizli O Penner IK Yonekawa T Naegelin Y Kuhle J Pardini M The association between olfactory bulb volume, cognitive dysfunction, physical disability and depression in multiple sclerosis. Eur J Neurol (2016) 23(3):510–9. 10.1111/ene.12891 26699999

[46] Calabrese M Atzori M Bernardi V Morra A Romualdi C Rinaldi L Cortical atrophy is relevant in multiple sclerosis at clinical onset. J Neurol (2007) 254(9):1212–20. 10.1007/s00415-006-0503-6 17361339

[47] Caramanos Z Francis SJ Narayanan S Lapierre Y Arnold DL Large, nonplateauing relationship between clinical disability and cerebral white matter lesion load in patients with multiple sclerosis. Arch Neurol (2012) 69(1):89–95. 10.1001/archneurol.2011.765 22232348

[48] Ramasamy DP Benedict RH Cox JL Fritz D Abdelrahman N Hussein S Extent of cerebellum, subcortical and cortical atrophy in patients with MS: a case-control study. J Neurol Sci (2009) 282(1–2):47–54. 10.1016/j.jns.2008.12.034 19201003

[49] van de Pavert SH Muhlert N Sethi V Wheeler-Kingshott CA Ridgway GR Geurts JJ DIR-visible grey matter lesions and atrophy in multiple sclerosis: partners in crime? J Neurol Neurosurg Psychiatry (2016) 87(5):461–7. 10.1136/jnnp-2014-310142 25926483 PMC4853554

[50] Motl RW Zivadinov R Bergsland N Benedict RH Thalamus volume and ambulation in multiple sclerosis: a cross-sectional study. Neurodegener Dis Manag (2016) 6(1):23–9. 10.2217/nmt.15.71 26782314

[51] Anderson VM Fisniku LK Altmann DR Thompson AJ Miller DH MRI measures show significant cerebellar gray matter volume loss in multiple sclerosis and are associated with cerebellar dysfunction. Mult Scler (2009) 15(7):811–7. 10.1177/1352458508101934 19465449

[52] Motl RW Hubbard EA Sreekumar N Wetter NC Sutton BP Pilutti LA Pallidal and caudate volumes correlate with walking function in multiple sclerosis. J Neurol Sci (2015) 354(1–2):33–6. 10.1016/j.jns.2015.04.041 25959979

[53] Shiee N Bazin PL Zackowski KM Farrell SK Harrison DM Newsome SD Revisiting brain atrophy and its relationship to disability in multiple sclerosis. PLoS One (2012) 7(5):e37049 10.1371/journal.pone.0037049 22615886 PMC3352847

[54] Jaworski J Psujek M Janczarek M Szczerbo-Trojanowska M Bartosik-Psujek H Total-tau in cerebrospinal fluid of patients with multiple sclerosis decreases in secondary progressive stage of disease and reflects degree of brain atrophy. Ups J Med Sci (2012) 117(3):284–92. 10.3109/03009734.2012.669423 22554142 PMC3410288

[55] Thaler C Faizy T Sedlacik J Holst B Stellmann JP Young KL T1-thresholds in black holes increase clinical-radiological correlation in multiple sclerosis patients. PLoS One (2015) 10(12):e0144693 10.1371/journal.pone.0144693 26659852 PMC4676682

[56] Granberg T Bergendal G Shams S Aspelin P Kristoffersen-Wiberg M Fredrikson S MRI-defined corpus callosal atrophy in multiple sclerosis: a comparison of volumetric measurements, corpus callosum area and index. J Neuroimaging (2015) 25(6):996–1001. 10.1111/jon.12237 25786805

[57] Sbardella E Petsas N Tona F Prosperini L Raz E Pace G Assessing the correlation between grey and white matter damage with motor and cognitive impairment in multiple sclerosis patients. PLoS One (2013) 8(5):e63250 10.1371/journal.pone.0063250 23696802 PMC3655958

[58] Chu R Tauhid S Glanz BI Healy BC Kim G Oommen VV Whole brain volume measured from 1.5T versus 3T MRI in healthy subjects and patients with multiple sclerosis. J Neuroimaging (2016) 26(1):62–7. 10.1111/jon.12271 26118637 PMC4755143

[59] Tam RC Traboulsee A Riddehough A Sheikhzadeh F Li DK The impact of intensity variations in T1-hypointense lesions on clinical correlations in multiple sclerosis. Mult Scler (2011) 17(8):949–57. 10.1177/1352458511402113 21502309

[60] Zimmermann H Rolfsnes HO Montag S Wilting J Droby A Reuter E Putaminal alteration in multiple sclerosis patients with spinal cord lesions. J Neural Transm (2015) 122(10):1465–73. 10.1007/s00702-015-1406-4 25971605

[61] Gorgoraptis N Wheeler-Kingshott CA Jenkins TM Altmann DR Miller DH Thompson AJ Combining tractography and cortical measures to test system-specific hypotheses in multiple sclerosis. Mult Scler (2010) 16(5):555–65. 10.1177/1352458510362440 20215478 PMC2925387

[62] Varoglu AO Odaci E Gumus H Keles ON Unal B Deniz O. Evaluation of patients with multiple sclerosis using a combination of morphometrical features and clinical scores. J Clin Neurosci (2010) 17(2):191–5. 10.1016/j.jocn.2009.04.023 20036126

[63] Anderson VM Wheeler-Kingshott CA Abdel-Aziz K Miller DH Toosy A Thompson AJ A comprehensive assessment of cerebellar damage in multiple sclerosis using diffusion tractography and volumetric analysis. Mult Scler (2011) 17(9):1079–87. 10.1177/1352458511403528 21511688 PMC3281565

[64] Hofstetter L Naegelin Y Filli L Kuster P Traud S Smieskova R Progression in disability and regional grey matter atrophy in relapsing-remitting multiple sclerosis. Mult Scler (2014) 20(2):202–13. 10.1177/1352458513493034 23804554 PMC6534500

[65] Vaneckova M Seidl Z Krasensky J Havrdova E Horakova D Dolezal O Patients’ stratification and correlation of brain magnetic resonance imaging parameters with disability progression in multiple sclerosis. Eur Neurol (2009) 61(5):278–84. 10.1159/000206852 19295214

[66] Giorgio A Stromillo ML Bartolozzi ML Rossi F Battaglini M De Leucio A Relevance of hypointense brain MRI lesions for long-term worsening of clinical disability in relapsing multiple sclerosis. Mult Scler (2014) 20(2):214–9. 10.1177/1352458513494490 23877971

[67] Rocca MA Mesaros S Pagani E Sormani MP Comi G Filippi M Thalamic damage and long-term progression of disability in multiple sclerosis. Radiology (2010) 257(2):463–9. 10.1148/radiol.10100326 20724544

[68] Mesaros S Rocca MA Pagani E Sormani MP Petrolini M Comi G Thalamic damage predicts the evolution of primary-progressive multiple sclerosis at 5 years. AJNR Am J Neuroradiol (2011) 32(6):1016–20. 10.3174/ajnr.A2430 21393412 PMC8013161

[69] Eshaghi A Bodini B Ridgway GR Garcia-Lorenzo D Tozer DJ Sahraian MA Temporal and spatial evolution of grey matter atrophy in primary progressive multiple sclerosis. Neuroimage (2014) 86:257–64. 10.1016/j.neuroimage.2013.09.059 24099844 PMC3898881

[70] Tedeschi G Dinacci D Comerci M Lavorgna L Savettieri G Quattrone A Brain atrophy evolution and lesion load accrual in multiple sclerosis: a 2-year follow-up study. Mult Scler (2009) 15(2):204–11. 10.1177/1352458508098270 18987104

[71] Gauthier SA Mandel M Guttmann CR Glanz BI Khoury SJ Betensky RA Predicting short-term disability in multiple sclerosis. Neurology (2007) 68(24):2059–65. 10.1212/01.wnl.0000264890.97479.b1 17562826

[72] Yaldizli O Atefy R Gass A Sturm D Glassl S Tettenborn B Corpus callosum index and long-term disability in multiple sclerosis patients. J Neurol (2010) 257(8):1256–64. 10.1007/s00415-010-5503-x 20198382

[73] Figueira FF Santos VS Figueira GM Silva AC Corpus callosum index: a practical method for long-term follow-up in multiple sclerosis. Arq Neuropsiquiatr (2007) 65(4a):931–5. 10.1590/S0004-282X2007000600001 18094848

[74] Neema M Arora A Healy BC Guss ZD Brass SD Duan Y Deep gray matter involvement on brain MRI scans is associated with clinical progression in multiple sclerosis. J Neuroimaging (2009) 19(1):3–8. 10.1111/j.1552-6569.2008.00296.x 19192042 PMC2762230

[75] Minneboo A Jasperse B Barkhof F Uitdehaag BM Knol DL de Groot V Predicting short-term disability progression in early multiple sclerosis: added value of MRI parameters. J Neurol Neurosurg Psychiatry (2008) 79(8):917–23. 10.1136/jnnp.2007.124123 18077480

[76] Moodie J Healy BC Buckle GJ Gauthier SA Glanz BI Arora A Magnetic Resonance Disease Severity Scale (MRDSS) for patients with multiple sclerosis: a longitudinal study. J Neurol Sci (2012) 315(1–2):49–54. 10.1016/j.jns.2011.11.040 22209496 PMC3319060

[77] Jacobsen C Hagemeier J Myhr KM Nyland H Lode K Bergsland N Brain atrophy and disability progression in multiple sclerosis patients: a 10-year follow-up study. J Neurol Neurosurg Psychiatry (2014) 85(10):1109–15. 10.1136/jnnp-2013-306906 24554101

[78] Filippi M Preziosa P Copetti M Riccitelli G Horsfield MA Martinelli V Gray matter damage predicts the accumulation of disability 13 years later in MS. Neurology (2013) 81(20):1759–67. 10.1212/01.wnl.0000435551.90824.d0 24122185

[79] Fisher E Lee JC Nakamura K Rudick RA Gray matter atrophy in multiple sclerosis: a longitudinal study. Ann Neurol (2008) 64(3):255–65. 10.1002/ana.21436 18661561

[80] Rudick RA Lee JC Nakamura K Fisher E Gray matter atrophy correlates with MS disability progression measured with MSFC but not EDSS. J Neurol Sci (2009) 282(1–2):106–11. 10.1016/j.jns.2008.11.018 19100997 PMC2726444

[81] Minneboo A Uitdehaag BM Jongen P Vrenken H Knol D van Walderveen MA Association between MRI parameters and the MS severity scale: a 12 year follow-up study. Mult Scler (2009) 15(5):632–7. 10.1177/1352458509102617 19389751

[82] Martola J Stawiarz L Fredrikson S Hillert J Bergstrom J Flodmark O Progression of non-age-related callosal brain atrophy in multiple sclerosis: a 9-year longitudinal MRI study representing four decades of disease development. J Neurol Neurosurg Psychiatry (2007) 78(4):375–80. 10.1136/jnnp.2006.106690 17119006 PMC2077793

[83] Martola J Stawiarz L Fredrikson S Hillert J Bergstrom J Flodmark O Rate of ventricular enlargement in multiple sclerosis: a nine-year magnetic resonance imaging follow-up study. Acta Radiol (2008) 49(5):570–9. 10.1080/02841850802039898 18568545

[84] Martola J Bergstrom J Fredrikson S Stawiarz L Hillert J Zhang Y A longitudinal observational study of brain atrophy rate reflecting four decades of multiple sclerosis: a comparison of serial 1D, 2D, and volumetric measurements from MRI images. Neuroradiology (2010) 52(2):109–17. 10.1007/s00234-009-0593-9 19774369

[85] Pichler A Khalil M Langkammer C Pinter D Bachmaier G Ropele S Combined analysis of global and compartmental brain volume changes in early multiple sclerosis in clinical practice. Mult Scler (2016) 22(3):340–6. 10.1177/1352458515593405 26163072

[86] Masek M Vaneckova M Krasensky J Danes J Havrdova E Hrebikova T Secondary-progressive form of multiple sclerosis: MRI changes versus clinical status. Neuro Endocrinol Lett (2008) 29(4):461–6. 18766142

[87] Filippi M. MRI measures of neurodegeneration in multiple sclerosis: implications for disability, disease monitoring, and treatment. J Neurol (2015) 262(1):1–6. 10.1007/s00415-014-7340-9 24723117

[88] Kearney H Miller DH Ciccarelli O Spinal cord MRI in multiple sclerosis – diagnostic, prognostic and clinical value. Nat Rev Neurol (2015) 11(6):327–38. 10.1038/nrneurol.2015.80 26009002

[89] Valsasina P Rocca MA Horsfield MA Copetti M Filippi M A longitudinal MRI study of cervical cord atrophy in multiple sclerosis. J Neurol (2015) 262(7):1622–8. 10.1007/s00415-015-7754-z 25929665

[90] Bernitsas E Bao F Seraji-Bozorgzad N Chorostecki J Santiago C Tselis A Spinal cord atrophy in multiple sclerosis and relationship with disability across clinical phenotypes. Mult Scler Relat Disord (2015) 4(1):47–51. 10.1016/j.msard.2014.11.002 25787052

[91] Biberacher V Boucard CC Schmidt P Engl C Buck D Berthele A Atrophy and structural variability of the upper cervical cord in early multiple sclerosis. Mult Scler (2015) 21(7):875–84. 10.1177/1352458514546514 25139943

[92] Daams M Weiler F Steenwijk MD Hahn HK Geurts JJ Vrenken H Mean upper cervical cord area (MUCCA) measurement in long-standing multiple sclerosis: relation to brain findings and clinical disability. Mult Scler (2014) 20(14):1860–5. 10.1177/1352458514533399 24812042

[93] Horsfield MA Sala S Neema M Absinta M Bakshi A Sormani MP Rapid semi-automatic segmentation of the spinal cord from magnetic resonance images: application in multiple sclerosis. Neuroimage (2010) 50(2):446–55. 10.1016/j.neuroimage.2009.12.121 20060481 PMC2830007

[94] Oh J Seigo M Saidha S Sotirchos E Zackowski K Chen M Spinal cord normalization in multiple sclerosis. J Neuroimaging (2014) 24(6):577–84. 10.1111/jon.12097 24593281 PMC4156567

[95] Rocca MA Horsfield MA Sala S Copetti M Valsasina P Mesaros S A multicenter assessment of cervical cord atrophy among MS clinical phenotypes. Neurology (2011) 76(24):2096–102. 10.1212/WNL.0b013e31821f46b8 21670439

[96] Schlaeger R Papinutto N Panara V Bevan C Lobach IV Bucci M Spinal cord gray matter atrophy correlates with multiple sclerosis disability. Ann Neurol (2014) 76(4):568–80. 10.1002/ana.24241 25087920 PMC5316412

[97] Song F Huan Y Yin H Ge Y Wei G Chang Y Normalized upper cervical spinal cord atrophy in multiple sclerosis. J Neuroimaging (2008) 18(3):320–7. 10.1111/j.1552-6569.2007.00222.x 18318794

[98] Valsasina P Rocca MA Horsfield MA Absinta M Messina R Caputo D Regional cervical cord atrophy and disability in multiple sclerosis: a voxel-based analysis. Radiology (2013) 266(3):853–61. 10.1148/radiol.12120813 23192778

[99] Yiannakas MC Mustafa AM De Leener B Kearney H Tur C Altmann DR Fully automated segmentation of the cervical cord from T1-weighted MRI using PropSeg: application to multiple sclerosis. Neuroimage Clin (2016) 10:71–7. 10.1016/j.nicl.2015.11.001 26793433 PMC4678307

[100] Benedetti B Rocca MA Rovaris M Caputo D Zaffaroni M Capra R A diffusion tensor MRI study of cervical cord damage in benign and secondary progressive multiple sclerosis patients. J Neurol Neurosurg Psychiatry (2010) 81(1):26–30. 10.1136/jnnp.2009.173120 19546104

[101] Healy BC Arora A Hayden DL Ceccarelli A Tauhid SS Neema M Approaches to normalization of spinal cord volume: application to multiple sclerosis. J Neuroimaging (2012) 22(3):e12–9. 10.1111/j.1552-6569.2011.00629.x 21854479 PMC3290735

[102] Weier K Mazraeh J Naegelin Y Thoeni A Hirsch JG Fabbro T Biplanar MRI for the assessment of the spinal cord in multiple sclerosis. Mult Scler (2012) 18(11):1560–9. 10.1177/1352458512442754 22539086

[103] Blamire AM Cader S Lee M Palace J Matthews PM Axonal damage in the spinal cord of multiple sclerosis patients detected by magnetic resonance spectroscopy. Magn Reson Med (2007) 58(5):880–5. 10.1002/mrm.21382 17969113

[104] Losseff NA Webb SL O’Riordan JI Page R Wang L Barker GJ Spinal cord atrophy and disability in multiple sclerosis. A new reproducible and sensitive MRI method with potential to monitor disease progression. Brain (1996) 119(Pt 3):701–8. 10.1093/brain/119.3.701 8673483

[105] Chen M Carass A Oh J Nair G Pham DL Reich DS Automatic magnetic resonance spinal cord segmentation with topology constraints for variable fields of view. Neuroimage (2013) 83:1051–62. 10.1016/j.neuroimage.2013.07.060 23927903 PMC3823375

[106] Rocca MA Valsasina P Damjanovic D Horsfield MA Mesaros S Stosic-Opincal T Voxel-wise mapping of cervical cord damage in multiple sclerosis patients with different clinical phenotypes. J Neurol Neurosurg Psychiatry (2013) 84(1):35–41. 10.1136/jnnp-2012-303821 23064100

[107] Lin X Blumhardt LD. Inflammation and atrophy in multiple sclerosis: MRI associations with disease course. J Neurol Sci (2001) 189(1–2):99–104. 10.1016/S0022-510X(01)00576-7 11535239

[108] Schlaeger R Papinutto N Zhu AH Lobach IV Bevan CJ Bucci M Association between thoracic spinal cord gray matter atrophy and disability in multiple sclerosis. JAMA Neurol (2015) 72(8):897–904. 10.1001/jamaneurol.2015.0993 26053119 PMC6002864

[109] Lukas C Knol DL Sombekke MH Bellenberg B Hahn HK Popescu V Cervical spinal cord volume loss is related to clinical disability progression in multiple sclerosis. J Neurol Neurosurg Psychiatry (2015) 86(4):410–8. 10.1136/jnnp-2014-308021 24973341

[110] Lukas C Sombekke MH Bellenberg B Hahn HK Popescu V Bendfeldt K Relevance of spinal cord abnormalities to clinical disability in multiple sclerosis: MR imaging findings in a large cohort of patients. Radiology (2013) 269(2):542–52. 10.1148/radiol.13122566 23737540

[111] Bonati U Fisniku LK Altmann DR Yiannakas MC Furby J Thompson AJ Cervical cord and brain grey matter atrophy independently associate with long-term MS disability. J Neurol Neurosurg Psychiatry (2011) 82(4):471–2. 10.1136/jnnp.2010.205021 20710012

[112] Ruggieri S Petracca M Miller A Krieger S Ghassemi R Bencosme Y Association of deep gray matter damage with cortical and spinal cord degeneration in primary progressive multiple sclerosis. JAMA Neurol (2015) 72(12):1466–74. 10.1001/jamaneurol.2015.1897 26457955

[113] Kolind S Seddigh A Combes A Russell-Schulz B Tam R Yogendrakumar V Brain and cord myelin water imaging: a progressive multiple sclerosis biomarker. Neuroimage Clin (2015) 9:574–80. 10.1016/j.nicl.2015.10.002 26594633 PMC4625204

[114] Tench CR Morgan PS Constantinescu CS. Measurement of cervical spinal cord cross-sectional area by MRI using edge detection and partial volume correction. J Magn Reson Imaging (2005) 21(3):197–203. 10.1002/jmri.20253 15723367

[115] Furby J Hayton T Anderson V Altmann D Brenner R Chataway J Magnetic resonance imaging measures of brain and spinal cord atrophy correlate with clinical impairment in secondary progressive multiple sclerosis. Mult Scler (2008) 14(8):1068–75. 10.1177/1352458508093617 18632782

[116] Losseff NA Wang L Lai HM Yoo DS Gawne-Cain ML McDonald WI Progressive cerebral atrophy in multiple sclerosis. A serial MRI study. Brain (1996) 119(Pt 6):2009–19. 10.1093/brain/119.6.2009 9010005

[117] Kearney H Rocca MA Valsasina P Balk L Sastre-Garriga J Reinhardt J Magnetic resonance imaging correlates of physical disability in relapse onset multiple sclerosis of long disease duration. Mult Scler (2014) 20(1):72–80. 10.1177/1352458513492245 23812283 PMC4107776

[118] Oh J Sotirchos ES Saidha S Whetstone A Chen M Newsome SD Relationships between quantitative spinal cord MRI and retinal layers in multiple sclerosis. Neurology (2015) 84(7):720–8. 10.1212/WNL.0000000000001257 25609766 PMC4336102

[119] Kearney H Altmann DR Samson RS Yiannakas MC Wheeler-Kingshott CA Ciccarelli O Cervical cord lesion load is associated with disability independently from atrophy in MS. Neurology (2015) 84(4):367–73. 10.1212/WNL.0000000000001186 25540312

[120] Zivadinov R Banas AC Yella V Abdelrahman N Weinstock-Guttman B Dwyer MG Comparison of three different methods for measurement of cervical cord atrophy in multiple sclerosis. AJNR Am J Neuroradiol (2008) 29(2):319–25. 10.3174/ajnr.A0813 17974604 PMC8118969

[121] Liptak Z Berger AM Sampat MP Charil A Felsovalyi O Healy BC Medulla oblongata volume: a biomarker of spinal cord damage and disability in multiple sclerosis. AJNR Am J Neuroradiol (2008) 29(8):1465–70. 10.3174/ajnr.A1162 18556361 PMC8119054

[122] Liu Y Wang J Daams M Weiler F Hahn HK Duan Y Differential patterns of spinal cord and brain atrophy in NMO and MS. Neurology (2015) 84(14):1465–72. 10.1212/WNL.0000000000001441 25762714

[123] Cohen AB Neema M Arora A Dell’oglio E Benedict RH Tauhid S The relationships among MRI-defined spinal cord involvement, brain involvement, and disability in multiple sclerosis. J Neuroimaging (2012) 22(2):122–8. 10.1111/j.1552-6569.2011.00589.x 21447024 PMC3128174

[124] Furby J Hayton T Altmann D Brenner R Chataway J Smith KJ A longitudinal study of MRI-detected atrophy in secondary progressive multiple sclerosis. J Neurol (2010) 257(9):1508–16. 10.1007/s00415-010-5563-y 20437181

[125] Agosta F Absinta M Sormani MP Ghezzi A Bertolotto A Montanari E In vivo assessment of cervical cord damage in MS patients: a longitudinal diffusion tensor MRI study. Brain (2007) 130(Pt 8):2211–9. 10.1093/brain/awm110 17535835

[126] Filippi M Agosta F Imaging biomarkers in multiple sclerosis. J Magn Reson Imaging (2010) 31(4):770–88. 10.1002/jmri.22102 20373420

[127] Filippi M Agosta F Magnetization transfer MRI in multiple sclerosis. J Neuroimaging (2007) 17(Suppl 1):22s–6s. 10.1111/j.1552-6569.2007.00129.x 17425730

[128] Rovaris M Agosta F Pagani E Filippi M Diffusion tensor MR imaging. Neuroimaging Clin N Am (2009) 19(1):37–43. 10.1016/j.nic.2008.08.001 19064198

[129] Bakshi R Neema M Healy BC Liptak Z Betensky RA Buckle GJ Predicting clinical progression in multiple sclerosis with the Magnetic Resonance Disease Severity Scale. Arch Neurol (2008) 65(11):1449–53. 10.1001/archneur.65.11.1449 19001162 PMC2762216

[130] Bakshi R Neema M Tauhid S Healy BC Glanz BI Kim G An expanded composite scale of MRI-defined disease severity in multiple sclerosis: MRDSS2. Neuroreport (2014) 25(14):1156–61. 10.1097/WNR.0000000000000244 25100554 PMC4166046

[131] Pardini M Yaldizli O Sethi V Muhlert N Liu Z Samson RS Motor network efficiency and disability in multiple sclerosis. Neurology (2015) 85(13):1115–22. 10.1212/WNL.0000000000001970 26320199 PMC4603887

[132] Hasan KM Walimuni IS Abid H Wolinsky JS Narayana PA Multi-modal quantitative MRI investigation of brain tissue neurodegeneration in multiple sclerosis. J Magn Reson Imaging (2012) 35(6):1300–11. 10.1002/jmri.23539 22241681 PMC3330152

[133] Branger P Parienti JJ Sormani MP Defer G The effect of disease-modifying drugs on brain atrophy in relapsing-remitting multiple sclerosis: a meta-analysis. PLoS One (2016) 11(3):e0149685 10.1371/journal.pone.0149685 26983008 PMC4794160

[134] Sormani MP Arnold DL De Stefano N Treatment effect on brain atrophy correlates with treatment effect on disability in multiple sclerosis. Ann Neurol (2014) 75(1):43–9. 10.1002/ana.24018 24006277

[135] Meyer-Moock S Feng YS Maeurer M Dippel FW Kohlmann T Systematic literature review and validity evaluation of the Expanded Disability Status Scale (EDSS) and the Multiple Sclerosis Functional Composite (MSFC) in patients with multiple sclerosis. BMC Neurol (2014) 14:58 10.1186/1471-2377-14-58 24666846 PMC3986942

[136] Alroughani R Deleu D El Salem K Al-Hashel J Alexander KJ Abdelrazek MA A regional consensus recommendation on brain atrophy as an outcome measure in multiple sclerosis. BMC Neurol (2016) 16(1):240 10.1186/s12883-016-0762-5 27881095 PMC5121973

