# Supplementary material for: The Role of T1-Weighted Derived Measures of Neurodegeneration for Assessing Disability Progression in Multiple Sclerosis
Source: Front Neurol. 2017 Sep 4;8:433. doi: 10.3389/fneur.2017.00433 (PMC5591328; doi:10.3389/fneur.2017.00433)
Supplement: Supplementary file 1 [file Table_1.DOCX]

**SUPPLEMENTARY APPENDIX PubMed search terminology**

MS terms

“Multiple sclerosis” [Title]

MRI terms

“Magnetic resonance imaging” [all fields]

MRI [all fields]

Magnetic Resonance Imaging [MeSH term]

Neurodegeneration terms

Neurodegenerati* [all fields]

Atrophy [all fields]

Volume [all fields]

“Black holes” [all fields]

T1-hypointens* OR “T1 hypointens*” [all fields]

“Axon* loss” OR “Axon* injury”

Disability terms

Disability [all fields]

EDSS or “Expanded Disability Status Scale” [all fields]

MSFC or “Multiple Sclerosis Functional Composite” [all fields]

T25FW or “Timed 25-Foot Walk” [all fields]

9HPT or “9-Hole Peg Test” [all fields]

“Motor performance” or “motor function” [all fields]

Limiters

Human

English language

Previous 10 years

Exclusions

Studies evaluating the effect of disease-modifying therapies on MRI measures of neurodegeneration
